# Supplementary material for: SuperAnimal pretrained pose estimation models for behavioral analysis
Source: Nat Commun. 2024 Jun 21;15:5165. doi: 10.1038/s41467-024-48792-2 (PMC11192880; doi:10.1038/s41467-024-48792-2)
Supplement: Supplementary file 1 — Supplementary Information [file 41467_2024_48792_MOESM1_ESM.pdf]

Supplementary Materials

Supplementary Figures

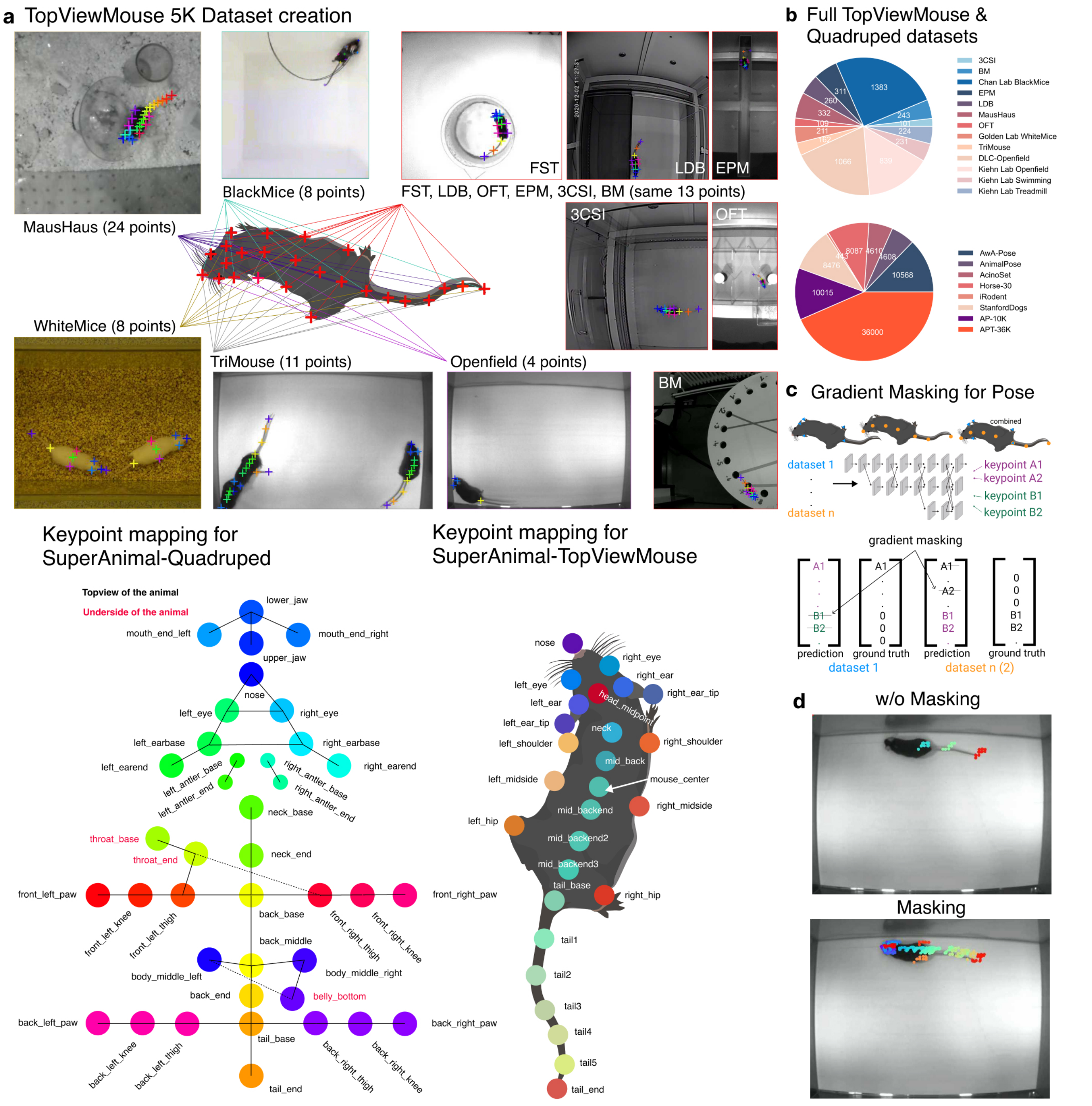

Figure S1. Constructing SuperAnimal models and keypoint gradient masking. **a**: Demonstration of how multiple pose datasets are merged into a single dataset. We created a main keypoint names to cover all keypoints we observe from datasets. Then we built a conversion table to map keypoints from each dataset to the main keypoint names. We design a corresponding conversion table such that anatomically similar keypoints are mapped to the same keypoint. Below we add the keypoint naming map for both SuperAnimal-TopViewMouse and SuperAnimal-Quadruped models. The mouse icons are modified from scidraw.io: https://beta.scidraw.io/drawing/183. Images in panel **a** labeled “Openfield" and “TriMouse" are adapted from `https://github.com/DeepLabCut/DeepLabCut` and are under a CC-BY license: `https://creativecommons.org/licenses/by/4.0/`; Image “MausHaus" is adapted from Mathis Laboratory of Adaptive Intelligence (2024) “MausHaus Mathis Lab”. Zenodo. `doi:10.5281/zenodo.10593101` and are under a CC-BY license: `https://creativecommons.org/licenses/by/4.0/`. **b**: Composition of the SuperAnimal-Quadruped (left) and SuperAnimal-TopViewMouse (right) datasets. **c**: Demonstration of keypoint gradient masking algorithm. Keypoints that were not defined in the original datasets introduce false penalties for the model training. Therefore, during back-propagation, the gradients of those undefined keypoints are artificially masked. **d**: With masking, the model is able to learn a pose representation that is the union of training datasets. Without masking, the model has severe degraded pose representation. Images in panel **d** are adapted from `https://github.com/DeepLabCut/DeepLabCut/blob/main/examples/openfield-Pranav-2018-10-30/videos/m3v1mp4.mp4` and are under a CC-BY license: `https://creativecommons.org/licenses/by/4.0/`.

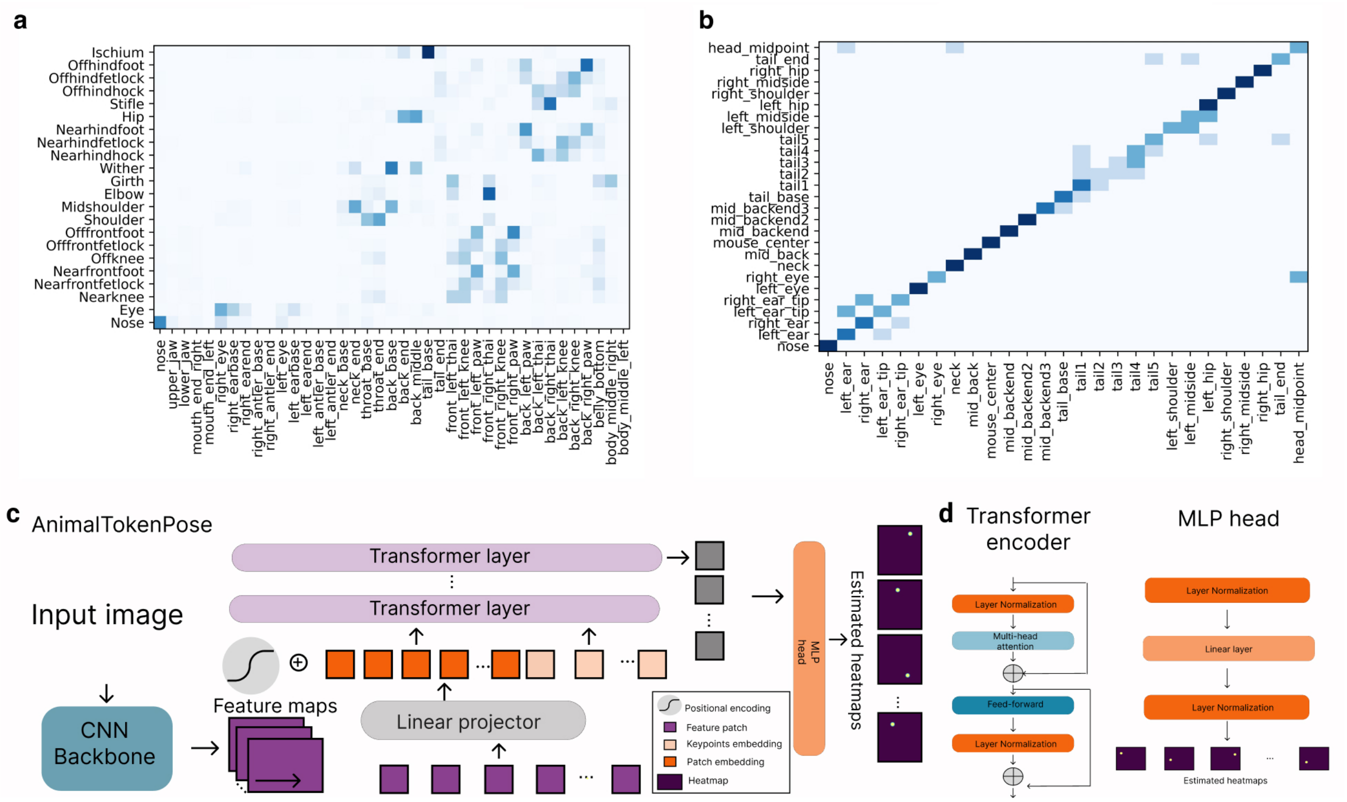

Figure S2. Keypoint Matching and AnimalTokenPose. **a**: The affinity matrix represents the semantic similarity between keypoint defined by the model and keypoint defined by dataset annotations across images. The affinity matrix is obtained by hard voting. The voting per image is obtained via pairwise euclidean distance between SuperAnimal-Quadruped model's zero-shot predictions and Horse-30 dataset ground truth. **b**: Affinity matrix for Golden Lab Mouse (see Methods) video (bottom at Figure 3), where we deliberately tried to match the keypoint space to model's zero-shot prediction. The noise in the affinity matrix suggests annotator bias for hard keypoints (e.g., tail points along the tail where the exact position is not visually concretely defined, as say opposed to the nose). For this analysis we annotated 20 frames of the Golden Lab Mouse data to illustrate our matching process. **c**: AnimalTokenPose architecture with additional MLP head for heatmap estimation. **d**: Transformer encoder architecture and MLP head architecture.

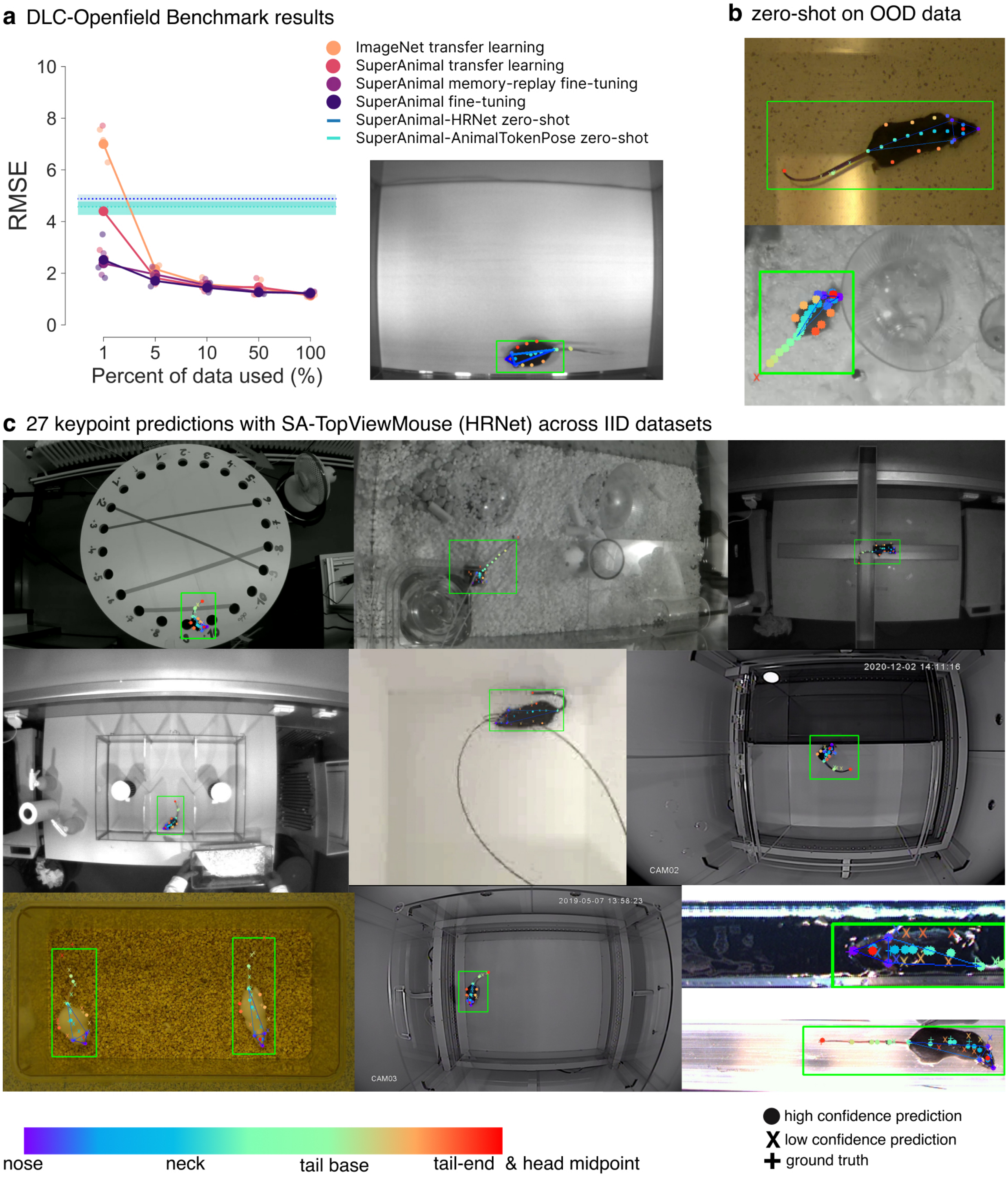

Figure S3. Top-down HRNet results a: SuperAnimal-TopViewMouse using HRNet-w32 on DLC Openfield benchmark. AnimalTokenPose is added as a zero-shot baseline. 1-100% of the train data is 10, 50, 101, 506, 1012 frames respectively. Blue shadow represents minimum, maximum and blue dash is the mean for zero-shot performance across three shuffles. Large, connected dots represent mean results across three shuffles and smaller dots represent results for individual shuffles. Inset is the qualitative zero-shot performance of SA-TVM. Inset image is adapted from `https://github.com/DeepLabCut/DeepLabCut/blob/main/examples/openfield-Pranav-2018-10-30/videos/m3v1mp4.mp4` and are under a CC-BY license: `https://creativecommons.org/licenses/by/4.0/`. **b**: Qualitative performance. SuperAnimal-TopViewMouse using HRNet on OOD videos (Top: Golden Lab; Bottom: Mathis MausHaus). Confidence cut off is set to be 0.6. **c** Qualitative performance. SuperAnimal-TopViewMouse using HRNet on IID images. Confidence cut off is set to be 0.6.

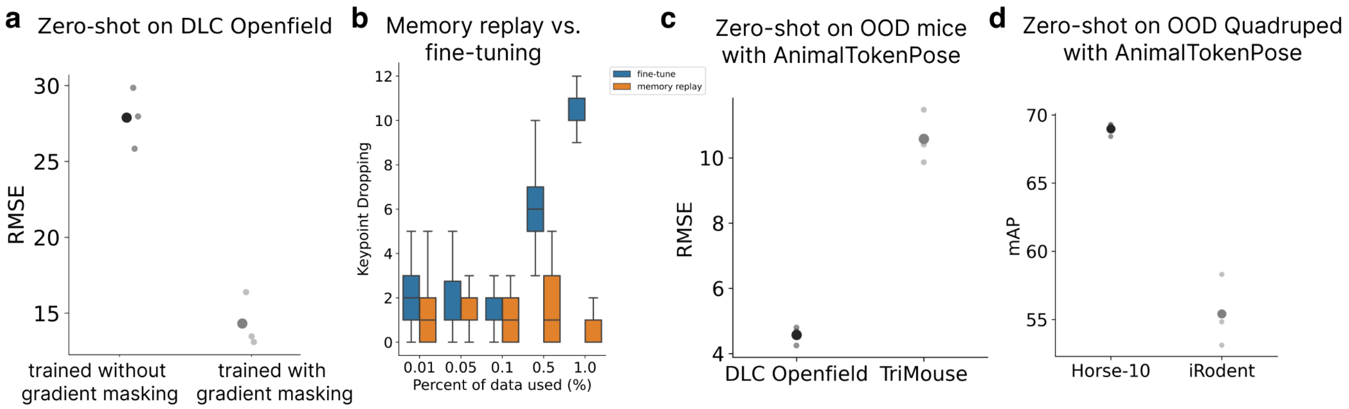

Figure S4. a: SA-TVM (DLCRNet) zero-shot performance on DLC Openfield. Comparison between SA-TVM trained with and without gradient masking. **b**: SA-TVM (HRNet-w32) fine-tuning performance on DLC Openfield. Comparison between SA-TVM fine-tuned with memory replay and naive fine-tuning across different training ratios. In box plots, the middle line indicates the median. The bounds of the box indicate the first and third quartiles and the whiskers extend to the farthest datapoint within 1.5 * IQR from the nearest hinge. **c** SuperAnimal-TopViewMouse using AnimalTokenPose. Zero-shot performance on DLC Openfield and TriMouse. **d** SuperAnimal-TopViewMouse using AnimalTokenPose. Zero-shot performance on iRodent and Horse-10.

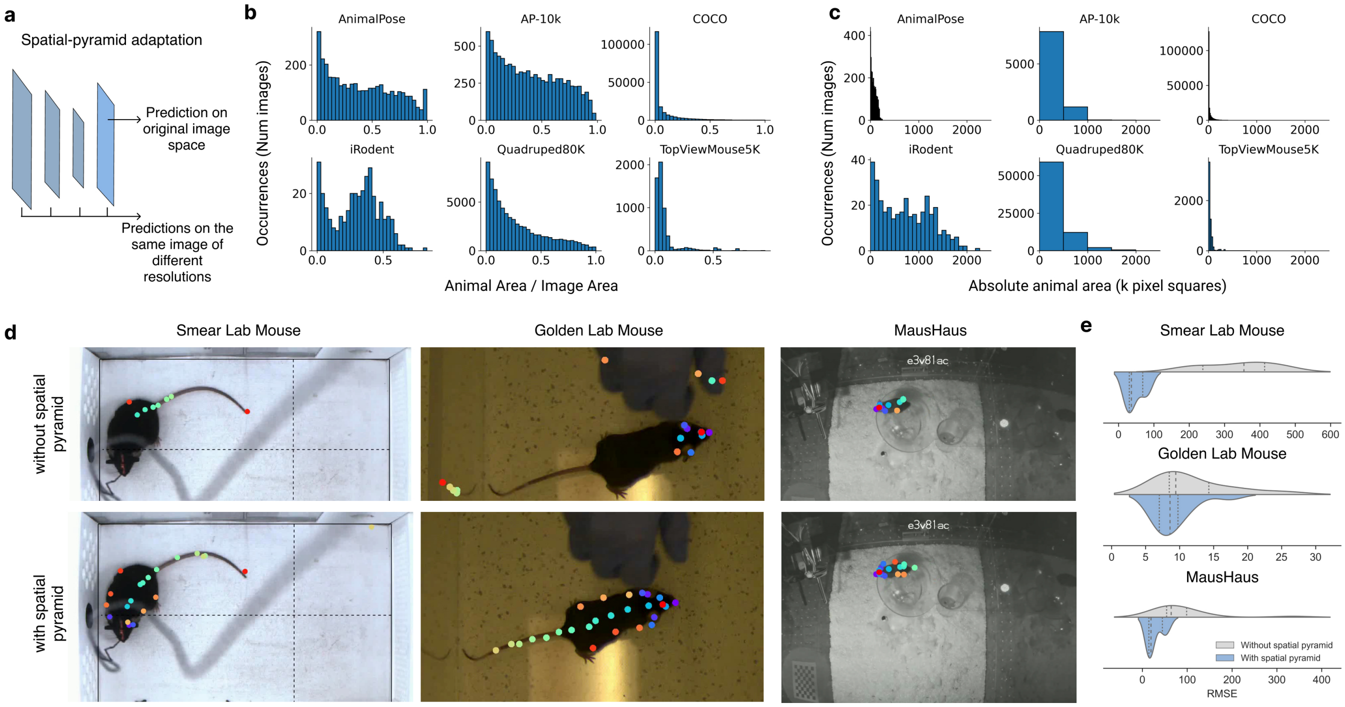

Figure S5. Challenges of animal appearance sizes a: Conceptual diagram to demonstrate that the spatial-pyramid search leverages prediction from multiple resolutions. **b**: Relative animal size with respect to the image size in common benchmarks. **c**: Absolute animal size (k pixel squares) in common benchmarks. **d**: Bottom-up SuperAnimal-TopViewMouse model (i.e., DLCRNet) was used to infer poses on three OOD videos. Visual inspection shows zero-shot inference with vs. without the spatial-pyramid search. **e**: Quantitative results between with and without spatial-pyramid adaptation on video frames from Smear Lab (n = 144 samples), Golden Lab (n = 4859 samples), and MausHaus (n = 3270 samples). Images on the far left are adapted from `https://edspace.american.edu/openbehavior/project/olfactory-search-video-donated-matt-smear/` and released under a CC BY-NC-SA license: `https://creativecommons.org/licenses/by-nc-sa/4.0/`. Images in the middle are adapted from `https://edspace.american.edu/openbehavior/project/open-field-social-investigation-videos-donated-sam-golden/` and released under a CC BY-NC-SA license: `https://creativecommons.org/licenses/by-nc-sa/4.0/`. Images on the far right are adapted from Mathis Laboratory of Adaptive Intelligence (2024) “MausHaus Mathis Lab”. Zenodo. `doi:10.5281/zenodo.10593101` and are under a CC-BY license: `https://creativecommons.org/licenses/by/4.0/`.

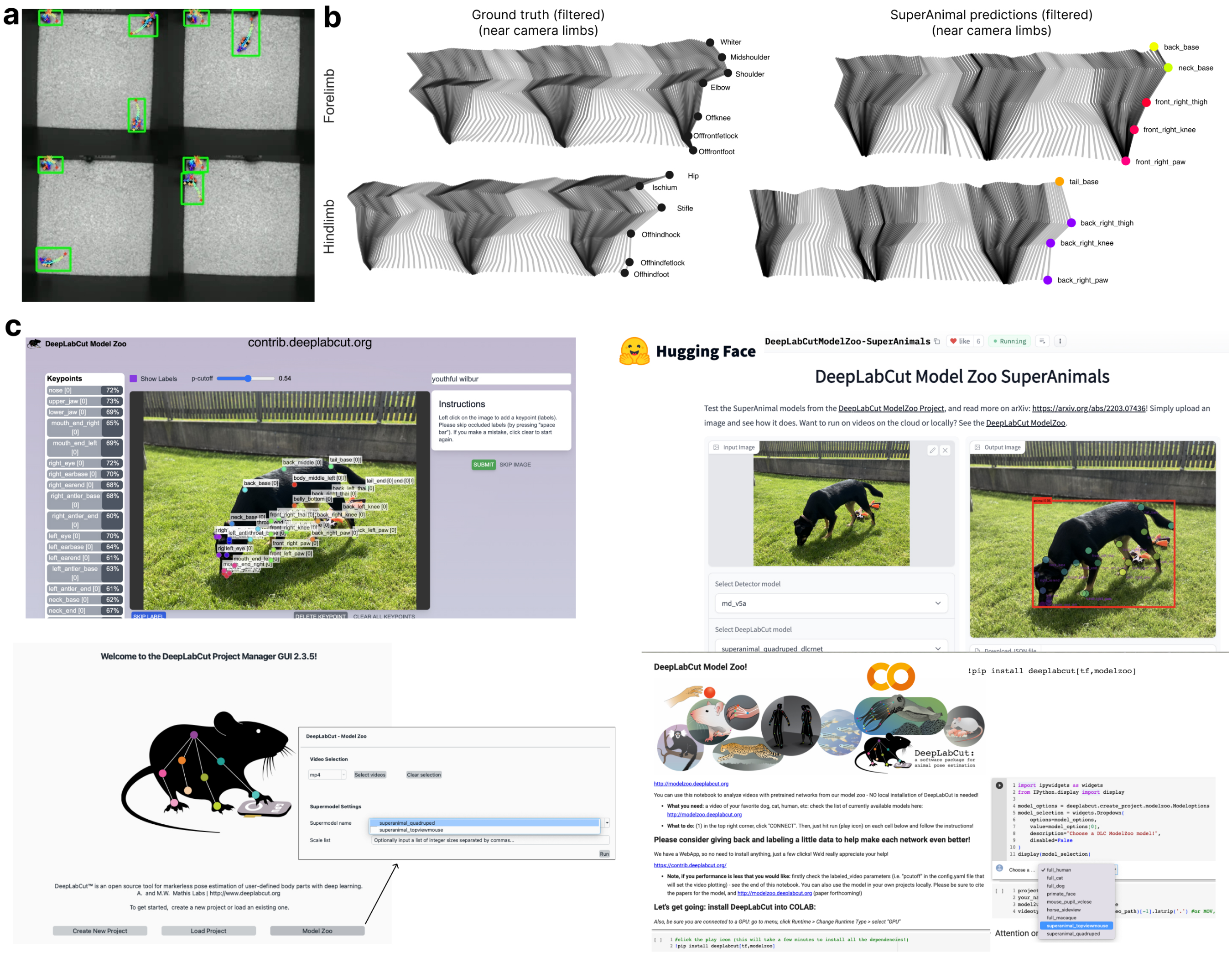

Figure S6. **a**: Visualization of top-down SuperAnimal-TopviewMouse on example MABe video frames, without trained on MABe videos. **b** Same as Figure 4g, but smoothed with a 3-Hz zero-lag, low-pass, 2nd order Butterworth filter. **c**: Top Left: An example of the current WebApp interface at `contrib.deeplabcut.org`. Users can add and edit the annotations from images we collect, following an anatomical figure that aids the expected location of bodyparts. Top Right: Example of current Gradio App on HuggingFace. Bottom Left: our current stand-alone GUI for local computer use showing a simple ModelZoo with SuperAnimal weights. Bottom Right: example of the Google Colaboratory interface with ModelZoo inference with SuperAnimal weights.

Supplementary Tables

Extended Full Results on Animal Benchmarks & Statistical Analysis

Table S1. Type-III Analysis of Variance Table for the mixed model relative to the quantification of memory replay in terms of keypoint dropping.

	Sum Sq	Mean Sq	NumDF	DenDF	F value	Pr(>F)
method	4096.16	4096.16	1.00	1608.00	2636.32	0.0000
train data_ratio	4839.77	1209.94	4.00	1608.00	778.73	0.0000
method: train data_ratio	5040.49	1260.12	4.00	1608.00	811.02	0.0000

Table S2. Two-sided pairwise contrasts adjusted with Tukey's method for the mixed model relative to the quantification of memory replay in terms of keypoint dropping

contrast	estimate	SE	df	t.ratio	p.value	eff.size
train data_ratio = 0.01						
fine-tune - (memory replay)	1.0309	0.1385	1608	7.443	<.0001	0.8270
train data_ratio = 0.05						
fine-tune - (memory replay)	0.3025	0.1385	1608	2.184	0.0291	0.2427
train data_ratio = 0.1						
fine-tune - (memory replay)	0.4444	0.1385	1608	3.209	0.0014	0.3566
train data_ratio = 0.5						
fine-tune - (memory replay)	4.6420	0.1385	1608	33.516	<.0001	3.7240
train data_ratio = 1						
fine-tune - (memory replay)	9.4815	0.1385	1608	68.459	<.0001	7.6065

Degrees-of-freedom method: kenward-roger

Table S3. HRNet-w32 TopViewMouse-5k **DLC Openfield**

method	pretrain_model	train data_ratio	mAP	RMSE
fine-tuning	SuperAnimal	0.01	98.813	2.518
fine-tuning	SuperAnimal	0.05	99.802	1.706
fine-tuning	SuperAnimal	0.1	99.892	1.439
fine-tuning	SuperAnimal	0.5	99.878	1.261
fine-tuning	SuperAnimal	1.0	99.925	1.234
memory replay	SuperAnimal	0.01	99.599	2.381
memory replay	SuperAnimal	0.05	99.765	1.954
memory replay	SuperAnimal	0.1	99.929	1.538
memory replay	SuperAnimal	0.5	99.778	1.293
memory replay	SuperAnimal	1.0	99.868	1.210
transfer learning	ImageNet	0.01	91.458	7.001
transfer learning	ImageNet	0.05	98.930	2.162
transfer learning	ImageNet	0.1	99.273	1.565
transfer learning	ImageNet	0.5	99.179	1.424
transfer learning	ImageNet	1.0	100.000	1.131
transfer learning	SuperAnimal	0.01	96.612	4.400
transfer learning	SuperAnimal	0.05	99.605	1.818
transfer learning	SuperAnimal	0.1	99.753	1.468
transfer learning	SuperAnimal	0.5	99.252	1.463
transfer learning	SuperAnimal	1.0	99.798	1.184
zero-shot	SuperAnimal	1.0	95.219	4.881

Table S4. HRNet-w32 TopViewMouse-5k **TriMouse**

method	pretrain_model	train data_ratio	mAP	RMSE
fine-tuning	SuperAnimal	0.01	88.516	9.196
fine-tuning	SuperAnimal	0.05	92.695	4.314
fine-tuning	SuperAnimal	0.1	97.543	2.865
fine-tuning	SuperAnimal	0.5	98.650	2.136
fine-tuning	SuperAnimal	1.0	99.021	2.020
memory replay	SuperAnimal	0.01	90.320	5.850
memory replay	SuperAnimal	0.05	93.569	4.188
memory replay	SuperAnimal	0.1	97.744	2.864
memory replay	SuperAnimal	0.5	98.618	2.184
memory replay	SuperAnimal	1.0	98.547	2.103
transfer learning	ImageNet	0.01	26.116	31.562
transfer learning	ImageNet	0.05	83.369	6.927
transfer learning	ImageNet	0.1	92.747	4.206
transfer learning	ImageNet	0.5	98.525	2.205
transfer learning	ImageNet	1.0	97.730	2.276
transfer learning	SuperAnimal	0.01	79.292	8.740
transfer learning	SuperAnimal	0.05	89.499	4.868
transfer learning	SuperAnimal	0.1	95.266	3.416
transfer learning	SuperAnimal	0.5	97.838	2.246
transfer learning	SuperAnimal	1.0	98.825	2.052
zero-shot	SuperAnimal	1.0	76.139	9.013

Table S5. Type-III Analysis of Variance Table for the top-down SuperAnimal-TopViewMouse **TriMouse** benchmark mixed model.

	Sum Sq	Mean Sq	NumDF	DenDF	F value	Pr(>F)
method	510.45	127.61	4.00	48.00	23.33	0.0000
data_ratio	927.34	231.83	4.00	48.00	42.39	0.0000
method:data_ratio	1180.53	73.78	16.00	48.00	13.49	0.0000

Table S6. Two-sided pairwise contrasts adjusted with Tukey's method for the top-down SuperAnimal-TopViewMouse **TriMouse** benchmark mixed model.

contrast	estimate	SE	df	t.ratio	p.value	eff.size
train data_ratio = 0.01						
(ImageNet transfer learning) - SuperAnimal fine-tune	22.3653	1.9095	48	11.713	<.0001	9.5633
(ImageNet transfer learning) - (SuperAnimal memory replay)	25.7114	1.9095	48	13.465	<.0001	10.9940
(ImageNet transfer learning) - (SuperAnimal transfer learning)	22.8212	1.9095	48	11.951	<.0001	9.7582
(ImageNet transfer learning) - SuperAnimal zero-shot	22.5486	1.9095	48	11.809	<.0001	9.6416
SuperAnimal fine-tune - (SuperAnimal memory replay)	3.3461	1.9095	48	1.752	0.4128	1.4308
SuperAnimal fine-tune - (SuperAnimal transfer learning)	0.4558	1.9095	48	0.239	0.9993	0.1949
SuperAnimal fine-tune - SuperAnimal zero-shot	0.1833	1.9095	48	0.096	1.0000	0.0784
(SuperAnimal memory replay) - (SuperAnimal transfer learning)	-2.8903	1.9095	48	-1.514	0.5589	-1.2359
(SuperAnimal memory replay) - SuperAnimal zero-shot	-3.1628	1.9095	48	-1.656	0.4700	-1.3524
(SuperAnimal transfer learning) - SuperAnimal zero-shot	-0.2726	1.9095	48	-0.143	0.9999	-0.1165
train data_ratio = 0.05						
(ImageNet transfer learning) - SuperAnimal fine-tune	2.6133	1.9095	48	1.369	0.6503	1.1174
(ImageNet transfer learning) - (SuperAnimal memory replay)	2.7392	1.9095	48	1.434	0.6089	1.1712
(ImageNet transfer learning) - (SuperAnimal transfer learning)	2.0592	1.9095	48	1.078	0.8167	0.8805
(ImageNet transfer learning) - SuperAnimal zero-shot	-2.0860	1.9095	48	-1.092	0.8095	-0.8920
SuperAnimal fine-tune - (SuperAnimal memory replay)	0.1259	1.9095	48	0.066	1.0000	0.0538
SuperAnimal fine-tune - (SuperAnimal transfer learning)	-0.5541	1.9095	48	-0.290	0.9984	-0.2369
SuperAnimal fine-tune - SuperAnimal zero-shot	-4.6993	1.9095	48	-2.461	0.1169	-2.0094
(SuperAnimal memory replay) - (SuperAnimal transfer learning)	-0.6800	1.9095	48	-0.356	0.9964	-0.2908
(SuperAnimal memory replay) - SuperAnimal zero-shot	-4.8252	1.9095	48	-2.527	0.1015	-2.0632
(SuperAnimal transfer learning) - SuperAnimal zero-shot	-4.1452	1.9095	48	-2.171	0.2083	-1.7725
train data_ratio = 0.1						
(ImageNet transfer learning) - SuperAnimal fine-tune	1.3415	1.9095	48	0.703	0.9549	0.5736

(ImageNet transfer learning) - (SuperAnimal memory replay)	1.3425	1.9095	48	0.703	0.9548	0.5740
(ImageNet transfer learning) - (SuperAnimal transfer learning)	0.7902	1.9095	48	0.414	0.9936	0.3379
(ImageNet transfer learning) - SuperAnimal zero-shot	-4.8068	1.9095	48	-2.517	0.1036	-2.0553
SuperAnimal fine-tune - (SuperAnimal memory replay)	0.0010	1.9095	48	0.000	1.0000	0.0004
SuperAnimal fine-tune - (SuperAnimal transfer learning)	-0.5513	1.9095	48	-0.289	0.9984	-0.2357
SuperAnimal fine-tune - SuperAnimal zero-shot	-6.1483	1.9095	48	-3.220	0.0186	-2.6290
(SuperAnimal memory replay) - (SuperAnimal transfer learning)	-0.5523	1.9095	48	-0.289	0.9984	-0.2361
(SuperAnimal memory replay) - SuperAnimal zero-shot	-6.1492	1.9095	48	-3.220	0.0186	-2.6294
(SuperAnimal transfer learning) - SuperAnimal zero-shot	-5.5970	1.9095	48	-2.931	0.0394	-2.3932
train data_ratio = 0.5						
(ImageNet transfer learning) - SuperAnimal fine-tune	0.0686	1.9095	48	0.036	1.0000	0.0293
(ImageNet transfer learning) - (SuperAnimal memory replay)	0.0210	1.9095	48	0.011	1.0000	0.0090
(ImageNet transfer learning) - (SuperAnimal transfer learning)	-0.0409	1.9095	48	-0.021	1.0000	-0.0175
(ImageNet transfer learning) - SuperAnimal zero-shot	-6.8083	1.9095	48	-3.565	0.0071	-2.9112
SuperAnimal fine-tune - (SuperAnimal memory replay)	-0.0477	1.9095	48	-0.025	1.0000	-0.0204
SuperAnimal fine-tune - (SuperAnimal transfer learning)	-0.1095	1.9095	48	-0.057	1.0000	-0.0468
SuperAnimal fine-tune - SuperAnimal zero-shot	-6.8769	1.9095	48	-3.601	0.0064	-2.9405
(SuperAnimal memory replay) - (SuperAnimal transfer learning)	-0.0618	1.9095	48	-0.032	1.0000	-0.0264
(SuperAnimal memory replay) - SuperAnimal zero-shot	-6.8293	1.9095	48	-3.576	0.0069	-2.9201
(SuperAnimal transfer learning) - SuperAnimal zero-shot	-6.7674	1.9095	48	-3.544	0.0076	-2.8937
train data_ratio = 1						
(ImageNet transfer learning) - SuperAnimal fine-tune	0.2566	1.9095	48	0.134	0.9999	0.1097
(ImageNet transfer learning) - (SuperAnimal memory replay)	0.1731	1.9095	48	0.091	1.0000	0.0740
(ImageNet transfer learning) - (SuperAnimal transfer learning)	0.2240	1.9095	48	0.117	1.0000	0.0958
(ImageNet transfer learning) - SuperAnimal zero-shot	-6.7367	1.9095	48	-3.528	0.0079	-2.8806
SuperAnimal fine-tune - (SuperAnimal memory replay)	-0.0835	1.9095	48	-0.044	1.0000	-0.0357
SuperAnimal fine-tune - (SuperAnimal transfer learning)	-0.0327	1.9095	48	-0.017	1.0000	-0.0140
SuperAnimal fine-tune - SuperAnimal zero-shot	-6.9933	1.9095	48	-3.662	0.0054	-2.9903
(SuperAnimal memory replay) - (SuperAnimal transfer learning)	0.0509	1.9095	48	0.027	1.0000	0.0217
(SuperAnimal memory replay) - SuperAnimal zero-shot	-6.9098	1.9095	48	-3.619	0.0061	-2.9546
(SuperAnimal transfer learning) - SuperAnimal zero-shot	-6.9606	1.9095	48	-3.645	0.0057	-2.9763

Degrees-of-freedom method: kenward-roger

P value adjustment: tukey method for comparing a family of 5 estimates

Table S7. Type-III Analysis of Variance Table for bottom-up **DLC-Openfield** mixed model.

	Sum Sq	Mean Sq	NumDF	DenDF	F value	Pr(>F)
method	845.14	211.29	4.00	48.00	46.06	0.0000
train data_ratio	839.85	209.96	4.00	48.00	45.78	0.0000
method:train data_ratio	407.57	25.47	16.00	48.00	5.55	0.0000

Table S8. Two-sided pairwise contrasts adjusted with Tukey's method for the bottom-up **DLC-Openfield** mixed model.

contrast	estimate	SE	df	t.ratio	p.value	eff.size
train data_ratio = 0.01						
ImageNet transfer learning - (SuperAnimal memory replay)	10.4477	1.7487	48	5.975	<.0001	4.8783
ImageNet transfer learning - SuperAnimal fine-tune	6.5661	1.7487	48	3.755	0.0041	3.0659
ImageNet transfer learning - SuperAnimal transfer learning	0.7263	1.7487	48	0.415	0.9936	0.3391
ImageNet transfer learning - (zero-shot)	3.8184	1.7487	48	2.184	0.2034	1.7829
(SuperAnimal memory replay) - SuperAnimal fine-tune	-3.8816	1.7487	48	-2.220	0.1900	-1.8124
(SuperAnimal memory replay) - SuperAnimal transfer learning	-9.7214	1.7487	48	-5.559	<.0001	-4.5391
(SuperAnimal memory replay) - (zero-shot)	-6.6293	1.7487	48	-3.791	0.0037	-3.0954
SuperAnimal fine-tune - SuperAnimal transfer learning	-5.8398	1.7487	48	-3.340	0.0135	-2.7267
SuperAnimal fine-tune - (zero-shot)	-2.7477	1.7487	48	-1.571	0.5226	-1.2830
SuperAnimal transfer learning - (zero-shot)	3.0921	1.7487	48	1.768	0.4036	1.4438
train data_ratio = 0.05						
ImageNet transfer learning - (SuperAnimal memory replay)	6.4436	1.7487	48	3.685	0.0050	3.0087
ImageNet transfer learning - SuperAnimal fine-tune	4.5692	1.7487	48	2.613	0.0839	2.1335
ImageNet transfer learning - SuperAnimal transfer learning	-0.0791	1.7487	48	-0.045	1.0000	-0.0370
ImageNet transfer learning - (zero-shot)	-2.5675	1.7487	48	-1.468	0.5876	-1.1988
(SuperAnimal memory replay) - SuperAnimal fine-tune	-1.8744	1.7487	48	-1.072	0.8199	-0.8752
(SuperAnimal memory replay) - SuperAnimal transfer learning	-6.5228	1.7487	48	-3.730	0.0044	-3.0456
(SuperAnimal memory replay) - (zero-shot)	-9.0111	1.7487	48	-5.153	<.0001	-4.2075
SuperAnimal fine-tune - SuperAnimal transfer learning	-4.6484	1.7487	48	-2.658	0.0757	-2.1704
SuperAnimal fine-tune - (zero-shot)	-7.1367	1.7487	48	-4.081	0.0015	-3.3323
SuperAnimal transfer learning - (zero-shot)	-2.4883	1.7487	48	-1.423	0.6162	-1.1619
train data_ratio = 0.1						
ImageNet transfer learning - (SuperAnimal memory replay)	2.1848	1.7487	48	1.249	0.7227	1.0201
ImageNet transfer learning - SuperAnimal fine-tune	2.8322	1.7487	48	1.620	0.4925	1.3224
ImageNet transfer learning - SuperAnimal transfer learning	1.3784	1.7487	48	0.788	0.9328	0.6436
ImageNet transfer learning - (zero-shot)	-8.0313	1.7487	48	-4.593	0.0003	-3.7500
(SuperAnimal memory replay) - SuperAnimal fine-tune	0.6474	1.7487	48	0.370	0.9959	0.3023
(SuperAnimal memory replay) - SuperAnimal transfer learning	-0.8064	1.7487	48	-0.461	0.9904	-0.3765
(SuperAnimal memory replay) - (zero-shot)	-10.2161	1.7487	48	-5.842	<.0001	-4.7701
SuperAnimal fine-tune - SuperAnimal transfer learning	-1.4538	1.7487	48	-0.831	0.9195	-0.6788
SuperAnimal fine-tune - (zero-shot)	-10.8635	1.7487	48	-6.212	<.0001	-5.0724
SuperAnimal transfer learning - (zero-shot)	-9.4097	1.7487	48	-5.381	<.0001	-4.3936
train data_ratio = 0.5						
ImageNet transfer learning - (SuperAnimal memory replay)	-0.5671	1.7487	48	-0.324	0.9975	-0.2648
ImageNet transfer learning - SuperAnimal fine-tune	-0.3719	1.7487	48	-0.213	0.9995	-0.1736
ImageNet transfer learning - SuperAnimal transfer learning	-0.2698	1.7487	48	-0.154	0.9999	-0.1260
ImageNet transfer learning - (zero-shot)	-11.6266	1.7487	48	-6.649	<.0001	-5.4287
(SuperAnimal memory replay) - SuperAnimal fine-tune	0.1952	1.7487	48	0.112	1.0000	0.0912
(SuperAnimal memory replay) - SuperAnimal transfer learning	0.2973	1.7487	48	0.170	0.9998	0.1388
(SuperAnimal memory replay) - (zero-shot)	-11.0595	1.7487	48	-6.325	<.0001	-5.1639
SuperAnimal fine-tune - SuperAnimal transfer learning	0.1021	1.7487	48	0.058	1.0000	0.0477
SuperAnimal fine-tune - (zero-shot)	-11.2548	1.7487	48	-6.436	<.0001	-5.2551
SuperAnimal transfer learning - (zero-shot)	-11.3568	1.7487	48	-6.495	<.0001	-5.3028
train data_ratio = 1.0						
ImageNet transfer learning - (SuperAnimal memory replay)	-0.7258	1.7487	48	-0.415	0.9936	-0.3389
ImageNet transfer learning - SuperAnimal fine-tune	-0.6376	1.7487	48	-0.365	0.9961	-0.2977
ImageNet transfer learning - SuperAnimal transfer learning	-0.4115	1.7487	48	-0.235	0.9993	-0.1921
ImageNet transfer learning - (zero-shot)	-11.9770	1.7487	48	-6.849	<.0001	-5.5923
(SuperAnimal memory replay) - SuperAnimal fine-tune	0.0882	1.7487	48	0.050	1.0000	0.0412
(SuperAnimal memory replay) - SuperAnimal transfer learning	0.3143	1.7487	48	0.180	0.9998	0.1468
(SuperAnimal memory replay) - (zero-shot)	-11.2512	1.7487	48	-6.434	<.0001	-5.2535
SuperAnimal fine-tune - SuperAnimal transfer learning	0.2261	1.7487	48	0.129	0.9999	0.1056
SuperAnimal fine-tune - (zero-shot)	-11.3395	1.7487	48	-6.485	<.0001	-5.2946
SuperAnimal transfer learning - (zero-shot)	-11.5656	1.7487	48	-6.614	<.0001	-5.4002

Degrees-of-freedom method: kenward-roger

P value adjustment: tukey method for comparing a family of 5 estimates

Table S9. Type-III Analysis of Variance Table for the top-down SuperAnimal-TopViewMouse DLC-Openfield benchmark mixed model.

	Sum Sq	Mean Sq	NumDF	DenDF	F value	Pr(>F)
method	109.24	27.31	4.00	48.00	74.19	0.0000
data_ratio	54.02	13.51	4.00	48.00	36.69	0.0000
method:data_ratio	45.72	2.86	16.00	48.00	7.76	0.0000

Table S10. Two-sided pairwise contrasts adjusted with Tukey's method for the top-down SuperAnimal-TopViewMouse DLC-Openfield benchmark mixed model.

contrast	estimate	SE	df	t.ratio	p.value	eff.size
train data_ratio = 0.01						
(ImageNet transfer learning) - SuperAnimal fine-tune	4.4830	0.4954	48	9.050	<.0001	7.3890
(ImageNet transfer learning) - (SuperAnimal memory replay)	4.6197	0.4954	48	9.326	<.0001	7.6143
(ImageNet transfer learning) - (SuperAnimal transfer learning)	2.6006	0.4954	48	5.250	<.0001	4.2864
(ImageNet transfer learning) - SuperAnimal zero-shot	2.1198	0.4954	48	4.279	0.0008	3.4940
SuperAnimal fine-tune - (SuperAnimal memory replay)	0.1367	0.4954	48	0.276	0.9987	0.2254
SuperAnimal fine-tune - (SuperAnimal transfer learning)	-1.8824	0.4954	48	-3.800	0.0036	-3.1026
SuperAnimal fine-tune - SuperAnimal zero-shot	-2.3631	0.4954	48	-4.770	0.0002	-3.8950
(SuperAnimal memory replay) - (SuperAnimal transfer learning)	-2.0191	0.4954	48	-4.076	0.0015	-3.3279
(SuperAnimal memory replay) - SuperAnimal zero-shot	-2.4998	0.4954	48	-5.046	0.0001	-4.1203
(SuperAnimal transfer learning) - SuperAnimal zero-shot	-0.4808	0.4954	48	-0.970	0.8670	-0.7924
train data_ratio = 0.05						
(ImageNet transfer learning) - SuperAnimal fine-tune	0.4564	0.4954	48	0.921	0.8873	0.7522
(ImageNet transfer learning) - (SuperAnimal memory replay)	0.2083	0.4954	48	0.421	0.9932	0.3434
(ImageNet transfer learning) - (SuperAnimal transfer learning)	0.3444	0.4954	48	0.695	0.9566	0.5677
(ImageNet transfer learning) - SuperAnimal zero-shot	-2.7190	0.4954	48	-5.489	<.0001	-4.4816
SuperAnimal fine-tune - (SuperAnimal memory replay)	-0.2480	0.4954	48	-0.501	0.9869	-0.4088
SuperAnimal fine-tune - (SuperAnimal transfer learning)	-0.1120	0.4954	48	-0.226	0.9994	-0.1845
SuperAnimal fine-tune - SuperAnimal zero-shot	-3.1754	0.4954	48	-6.410	<.0001	-5.2338
(SuperAnimal memory replay) - (SuperAnimal transfer learning)	0.1361	0.4954	48	0.275	0.9987	0.2243
(SuperAnimal memory replay) - SuperAnimal zero-shot	-2.9273	0.4954	48	-5.909	<.0001	-4.8250
(SuperAnimal transfer learning) - SuperAnimal zero-shot	-3.0634	0.4954	48	-6.184	<.0001	-5.0493
train data_ratio = 0.1						
(ImageNet transfer learning) - SuperAnimal fine-tune	0.1257	0.4954	48	0.254	0.9991	0.2072
(ImageNet transfer learning) - (SuperAnimal memory replay)	0.0271	0.4954	48	0.055	1.0000	0.0447
(ImageNet transfer learning) - (SuperAnimal transfer learning)	0.0972	0.4954	48	0.196	0.9997	0.1602
(ImageNet transfer learning) - SuperAnimal zero-shot	-3.3163	0.4954	48	-6.695	<.0001	-5.4661
SuperAnimal fine-tune - (SuperAnimal memory replay)	-0.0986	0.4954	48	-0.199	0.9996	-0.1625
SuperAnimal fine-tune - (SuperAnimal transfer learning)	-0.0285	0.4954	48	-0.058	1.0000	-0.0470
SuperAnimal fine-tune - SuperAnimal zero-shot	-3.4421	0.4954	48	-6.948	<.0001	-5.6733
(SuperAnimal memory replay) - (SuperAnimal transfer learning)	0.0701	0.4954	48	0.141	0.9999	0.1155
(SuperAnimal memory replay) - SuperAnimal zero-shot	-3.3435	0.4954	48	-6.749	<.0001	-5.5108
(SuperAnimal transfer learning) - SuperAnimal zero-shot	-3.4135	0.4954	48	-6.891	<.0001	-5.6263
train data_ratio = 0.5						
(ImageNet transfer learning) - SuperAnimal fine-tune	0.1628	0.4954	48	0.329	0.9974	0.2683
(ImageNet transfer learning) - (SuperAnimal memory replay)	0.1308	0.4954	48	0.264	0.9989	0.2155
(ImageNet transfer learning) - (SuperAnimal transfer learning)	-0.0392	0.4954	48	-0.079	1.0000	-0.0647
(ImageNet transfer learning) - SuperAnimal zero-shot	-3.4574	0.4954	48	-6.979	<.0001	-5.6986
SuperAnimal fine-tune - (SuperAnimal memory replay)	-0.0320	0.4954	48	-0.065	1.0000	-0.0528
SuperAnimal fine-tune - (SuperAnimal transfer learning)	-0.2020	0.4954	48	-0.408	0.9940	-0.3330
SuperAnimal fine-tune - SuperAnimal zero-shot	-3.6202	0.4954	48	-7.308	<.0001	-5.9669
(SuperAnimal memory replay) - (SuperAnimal transfer learning)	-0.1700	0.4954	48	-0.343	0.9969	-0.2802
(SuperAnimal memory replay) - SuperAnimal zero-shot	-3.5882	0.4954	48	-7.243	<.0001	-5.9141
(SuperAnimal transfer learning) - SuperAnimal zero-shot	-3.4182	0.4954	48	-6.900	<.0001	-5.6339
train data_ratio = 1						
(ImageNet transfer learning) - SuperAnimal fine-tune	-0.1033	0.4954	48	-0.208	0.9996	-0.1702
(ImageNet transfer learning) - (SuperAnimal memory replay)	-0.0790	0.4954	48	-0.159	0.9998	-0.1302
(ImageNet transfer learning) - (SuperAnimal transfer learning)	-0.0525	0.4954	48	-0.106	1.0000	-0.0865

Table S11. HRNet-w32 Quadruped80K **Horse-10**

method	pretrain_model	train data_ratio	mAP	NE_IID	NE_OOD	RMSE
fine-tuning	AP-10K	0.01	66.284	0.286	0.285	5.029
fine-tuning	AP-10K	0.05	80.265	0.187	0.187	2.950
fine-tuning	AP-10K	0.1	81.987	0.199	0.175	2.661
fine-tuning	AP-10K	0.5	91.369	0.070	0.101	1.557
fine-tuning	AP-10K	1.0	93.973	0.036	0.083	1.220
fine-tuning	SuperAnimal	0.01	71.684	0.219	0.213	3.855
fine-tuning	SuperAnimal	0.05	85.444	0.131	0.136	2.162
fine-tuning	SuperAnimal	0.1	88.787	0.113	0.121	1.885
fine-tuning	SuperAnimal	0.5	93.659	0.057	0.079	1.307
fine-tuning	SuperAnimal	1.0	95.433	0.038	0.073	1.133
memory replay	SuperAnimal	0.01	73.366	0.209	0.202	3.719
memory replay	SuperAnimal	0.05	83.762	0.140	0.146	2.426
memory replay	SuperAnimal	0.1	88.711	0.114	0.124	1.902
memory replay	SuperAnimal	0.5	93.555	0.060	0.083	1.366
memory replay	SuperAnimal	1.0	95.165	0.040	0.073	1.153
transfer learning	AP-10K	0.01	1.005	1.640	1.615	33.071
transfer learning	AP-10K	0.05	67.744	0.327	0.304	4.744
transfer learning	AP-10K	0.1	76.285	0.276	0.242	3.812
transfer learning	AP-10K	0.5	91.107	0.073	0.111	1.693
transfer learning	AP-10K	1.0	94.026	0.036	0.092	1.347
transfer learning	ImageNet	0.01	0.934	2.369	2.360	46.255
transfer learning	ImageNet	0.05	22.730	0.861	0.847	14.815
transfer learning	ImageNet	0.1	32.144	0.783	0.820	14.637
transfer learning	ImageNet	0.5	76.420	0.190	0.285	4.822
transfer learning	ImageNet	1.0	90.516	0.036	0.135	1.837
transfer learning	SuperAnimal	0.01	1.103	1.521	1.500	31.190
transfer learning	SuperAnimal	0.05	74.658	0.243	0.238	3.694
transfer learning	SuperAnimal	0.1	85.235	0.156	0.161	2.347
transfer learning	SuperAnimal	0.5	93.106	0.062	0.092	1.452
transfer learning	SuperAnimal	1.0	94.837	0.036	0.082	1.218
zero-shot	AP-10K	-	65.729	0.296	0.287	4.929
zero-shot	SuperAnimal	-	71.205	0.227	0.228	3.958

(ImageNet transfer learning) - SuperAnimal zero-shot	-3.7499	0.4954	48	-7.570	<.0001	-6.1807
SuperAnimal fine-tune - (SuperAnimal memory replay)	0.0243	0.4954	48	0.049	1.0000	0.0400
SuperAnimal fine-tune - (SuperAnimal transfer learning)	0.0508	0.4954	48	0.103	1.0000	0.0837
SuperAnimal fine-tune - SuperAnimal zero-shot	-3.6466	0.4954	48	-7.361	<.0001	-6.0105
(SuperAnimal memory replay) - (SuperAnimal transfer learning)	0.0265	0.4954	48	0.054	1.0000	0.0437
(SuperAnimal memory replay) - SuperAnimal zero-shot	-3.6709	0.4954	48	-7.410	<.0001	-6.0505
(SuperAnimal transfer learning) - SuperAnimal zero-shot	-3.6974	0.4954	48	-7.464	<.0001	-6.0942

Degrees-of-freedom method: kenward-roger, P value adjustment: tukey method for comparing a family of 5 estimates

Table S12. HRNet-w32 Quadruped80K **iRodent**

method	pretrain_model	train data_ratio	mAP	RMSE
fine-tuning	AP-10K	0.01	43.144	37.704
fine-tuning	AP-10K	0.05	49.605	34.235
fine-tuning	AP-10K	0.1	50.019	36.970
fine-tuning	AP-10K	0.5	57.858	29.547
fine-tuning	AP-10K	1.0	61.635	26.758
fine-tuning	SuperAnimal	0.01	59.194	32.599
fine-tuning	SuperAnimal	0.05	61.255	30.897
fine-tuning	SuperAnimal	0.1	61.042	34.594
fine-tuning	SuperAnimal	0.5	70.028	26.766
fine-tuning	SuperAnimal	1.0	72.247	25.065
memory replay	SuperAnimal	0.01	60.853	31.801
memory replay	SuperAnimal	0.05	63.275	29.757
memory replay	SuperAnimal	0.1	63.716	29.967
memory replay	SuperAnimal	0.5	69.263	26.188
memory replay	SuperAnimal	1.0	72.971	24.884
transfer learning	AP-10K	0.01	12.910	92.649
transfer learning	AP-10K	0.05	39.342	46.696
transfer learning	AP-10K	0.1	42.477	43.824
transfer learning	AP-10K	0.5	64.448	32.006
transfer learning	AP-10K	1.0	70.915	28.005
transfer learning	ImageNet	0.01	0.785	152.225
transfer learning	ImageNet	0.05	23.350	64.799
transfer learning	ImageNet	0.1	27.728	62.722
transfer learning	ImageNet	0.5	50.509	43.230
transfer learning	ImageNet	1.0	58.857	35.651
transfer learning	SuperAnimal	0.01	17.626	84.663
transfer learning	SuperAnimal	0.05	49.482	40.104
transfer learning	SuperAnimal	0.1	54.848	37.426
transfer learning	SuperAnimal	0.5	69.819	27.680
transfer learning	SuperAnimal	1.0	72.047	25.773
zero-shot	AP-10K	-	40.389	37.417
zero-shot	SuperAnimal	-	58.557	33.496

Table S13. HRNetw32 Quadruped80K **AnimalPose**

method	pretrain_model	train data_ratio	mAP	RMSE
fine-tuning	AP-10K	1.0	86.794	4.860
fine-tuning	SuperAnimal	1.0	86.851	4.706
memory replay	SuperAnimal	1.0	87.034	4.636
transfer learning	AP-10K	1.0	89.402	5.275
transfer learning	ImageNet	1.0	86.864	5.757
transfer learning	SuperAnimal	1.0	89.612	5.185
zero-shot	AP-10K	-	79.447	5.774
zero-shot	SuperAnimal	-	84.639	4.884

Table S14. HRNet-w32 Quadruped80K **AP-10K**

method	pretrain_model	train data_ratio	mAP	RMSE
fine-tuning	SuperAnimal	1.0	79.511	11.021
memory replay	SuperAnimal	1.0	80.113	11.296
transfer learning	ImageNet	1.0	70.548	11.228
transfer learning	SuperAnimal	1.0	74.379	10.748
zero-shot	SuperAnimal	-	68.038	12.971

Table S15. Type-III Analysis of Variance Table for Horse-10 OOD mixed model.

	Sum Sq	Mean Sq	NumDF	DenDF	F value	Pr(>F)
cond	2.10	0.30	7.00	78.00	183.97	0.0000
data_ratio	2.88	0.72	4.00	78.00	442.97	0.0000
cond:data_ratio	2.73	0.10	28.00	78.00	59.87	0.0000

Table S16. Two-sided pairwise contrasts adjusted with Tukey's method for the Horse-10 OOD mixed model.

contrast	estimate	SE	df	t.ratio	p.value	eff.size
train data_ratio = 0.01						
AP10k fine-tune - (AP10k transfer learning)	0.6528	0.0329	78	19.819	<.0001	16.1825
AP10k fine-tune - AP10k zero-shot	0.0055	0.0329	78	0.168	1.0000	0.1375
AP10k fine-tune - (ImageNet transfer learning)	0.6535	0.0329	78	19.841	<.0001	16.2000
AP10k fine-tune - SuperAnimal fine-tune	-0.0540	0.0329	78	-1.640	0.7249	-1.3388
AP10k fine-tune - (SuperAnimal memory replay)	-0.0708	0.0329	78	-2.150	0.3929	-1.7558
AP10k fine-tune - (SuperAnimal transfer learning)	0.6518	0.0329	78	19.790	<.0001	16.1581
AP10k fine-tune - SuperAnimal zero-shot	-0.0492	0.0329	78	-1.494	0.8082	-1.2200
(AP10k transfer learning) - AP10k zero-shot	-0.6472	0.0329	78	-19.651	<.0001	-16.0450
(AP10k transfer learning) - (ImageNet transfer learning)	0.0007	0.0329	78	0.021	1.0000	0.0175
(AP10k transfer learning) - SuperAnimal fine-tune	-0.7068	0.0329	78	-21.459	<.0001	-17.5213
(AP10k transfer learning) - (SuperAnimal memory replay)	-0.7236	0.0329	78	-21.970	<.0001	-17.9384
(AP10k transfer learning) - (SuperAnimal transfer learning)	-0.0010	0.0329	78	-0.030	1.0000	-0.0244
(AP10k transfer learning) - SuperAnimal zero-shot	-0.7020	0.0329	78	-21.314	<.0001	-17.4025
AP10k zero-shot - (ImageNet transfer learning)	0.6479	0.0329	78	19.673	<.0001	16.0625
AP10k zero-shot - SuperAnimal fine-tune	-0.0596	0.0329	78	-1.808	0.6166	-1.4763
AP10k zero-shot - (SuperAnimal memory replay)	-0.0764	0.0329	78	-2.319	0.2969	-1.8933
AP10k zero-shot - (SuperAnimal transfer learning)	0.6463	0.0329	78	19.621	<.0001	16.0206
AP10k zero-shot - SuperAnimal zero-shot	-0.0548	0.0329	78	-1.663	0.7108	-1.3575
(ImageNet transfer learning) - SuperAnimal fine-tune	-0.7075	0.0329	78	-21.481	<.0001	-17.5388
(ImageNet transfer learning) - (SuperAnimal memory replay)	-0.7243	0.0329	78	-21.991	<.0001	-17.9559
(ImageNet transfer learning) - (SuperAnimal transfer learning)	-0.0017	0.0329	78	-0.051	1.0000	-0.0419
(ImageNet transfer learning) - SuperAnimal zero-shot	-0.7027	0.0329	78	-21.335	<.0001	-17.4200
SuperAnimal fine-tune - (SuperAnimal memory replay)	-0.0168	0.0329	78	-0.511	0.9996	-0.4170
SuperAnimal fine-tune - (SuperAnimal transfer learning)	0.7058	0.0329	78	21.429	<.0001	17.4969
SuperAnimal fine-tune - SuperAnimal zero-shot	0.0048	0.0329	78	0.146	1.0000	0.1188
(SuperAnimal memory replay) - (SuperAnimal transfer learning)	0.7226	0.0329	78	21.940	<.0001	17.9140
(SuperAnimal memory replay) - SuperAnimal zero-shot	0.0216	0.0329	78	0.656	0.9978	0.5359
(SuperAnimal transfer learning) - SuperAnimal zero-shot	-0.7010	0.0329	78	-21.284	<.0001	-17.3781
train data_ratio = 0.05						
AP10k fine-tune - (AP10k transfer learning)	0.1252	0.0329	78	3.801	0.0066	3.1038
AP10k fine-tune - AP10k zero-shot	0.1454	0.0329	78	4.413	0.0008	3.6034
AP10k fine-tune - (ImageNet transfer learning)	0.5753	0.0329	78	17.468	<.0001	14.2628
AP10k fine-tune - SuperAnimal fine-tune	-0.0518	0.0329	78	-1.572	0.7650	-1.2839
AP10k fine-tune - (SuperAnimal memory replay)	-0.0350	0.0329	78	-1.062	0.9627	-0.8669
AP10k fine-tune - (SuperAnimal transfer learning)	0.0561	0.0329	78	1.702	0.6857	1.3899
AP10k fine-tune - SuperAnimal zero-shot	0.0906	0.0329	78	2.751	0.1232	2.2459
(AP10k transfer learning) - AP10k zero-shot	0.0202	0.0329	78	0.612	0.9986	0.4996
(AP10k transfer learning) - (ImageNet transfer learning)	0.4501	0.0329	78	13.667	<.0001	11.1590
(AP10k transfer learning) - SuperAnimal fine-tune	-0.1770	0.0329	78	-5.374	<.0001	-4.3877
(AP10k transfer learning) - (SuperAnimal memory replay)	-0.1602	0.0329	78	-4.863	0.0002	-3.9706
(AP10k transfer learning) - (SuperAnimal transfer learning)	-0.0691	0.0329	78	-2.099	0.4247	-1.7139
(AP10k transfer learning) - SuperAnimal zero-shot	-0.0346	0.0329	78	-1.051	0.9648	-0.8579
AP10k zero-shot - (ImageNet transfer learning)	0.4300	0.0329	78	13.055	<.0001	10.6594
AP10k zero-shot - SuperAnimal fine-tune	-0.1971	0.0329	78	-5.986	<.0001	-4.8873
AP10k zero-shot - (SuperAnimal memory replay)	-0.1803	0.0329	78	-5.475	<.0001	-4.4703
AP10k zero-shot - (SuperAnimal transfer learning)	-0.0893	0.0329	78	-2.711	0.1348	-2.2135
AP10k zero-shot - SuperAnimal zero-shot	-0.0548	0.0329	78	-1.663	0.7108	-1.3575
(ImageNet transfer learning) - SuperAnimal fine-tune	-0.6271	0.0329	78	-19.041	<.0001	-15.5467

(ImageNet transfer learning) - (SuperAnimal memory replay)	-0.6103	0.0329	78	-18.530	<.0001	-15.1297
(ImageNet transfer learning) - (SuperAnimal transfer learning)	-0.5193	0.0329	78	-15.766	<.0001	-12.8729
(ImageNet transfer learning) - SuperAnimal zero-shot	-0.4848	0.0329	78	-14.718	<.0001	-12.0169
SuperAnimal fine-tune - (SuperAnimal memory replay)	0.0168	0.0329	78	0.511	0.9996	0.4170
SuperAnimal fine-tune - (SuperAnimal transfer learning)	0.1079	0.0329	78	3.275	0.0323	2.6738
SuperAnimal fine-tune - SuperAnimal zero-shot	0.1424	0.0329	78	4.323	0.0011	3.5298
(SuperAnimal memory replay) - (SuperAnimal transfer learning)	0.0910	0.0329	78	2.764	0.1195	2.2568
(SuperAnimal memory replay) - SuperAnimal zero-shot	0.1256	0.0329	78	3.812	0.0064	3.1128
(SuperAnimal transfer learning) - SuperAnimal zero-shot	0.0345	0.0329	78	1.048	0.9652	0.8560
train data_ratio = 0.1						
AP10k fine-tune - (AP10k transfer learning)	0.0570	0.0329	78	1.731	0.6672	1.4135
AP10k fine-tune - AP10k zero-shot	0.1626	0.0329	78	4.936	0.0001	4.0304
AP10k fine-tune - (ImageNet transfer learning)	0.4984	0.0329	78	15.133	<.0001	12.3559
AP10k fine-tune - SuperAnimal fine-tune	-0.0680	0.0329	78	-2.064	0.4467	-1.6856
AP10k fine-tune - (SuperAnimal memory replay)	-0.0672	0.0329	78	-2.041	0.4616	-1.6667
AP10k fine-tune - (SuperAnimal transfer learning)	-0.0325	0.0329	78	-0.986	0.9752	-0.8050
AP10k fine-tune - SuperAnimal zero-shot	0.1078	0.0329	78	3.274	0.0324	2.6729
(AP10k transfer learning) - AP10k zero-shot	0.1056	0.0329	78	3.205	0.0392	2.6169
(AP10k transfer learning) - (ImageNet transfer learning)	0.4414	0.0329	78	13.402	<.0001	10.9424
(AP10k transfer learning) - SuperAnimal fine-tune	-0.1250	0.0329	78	-3.796	0.0067	-3.0991
(AP10k transfer learning) - (SuperAnimal memory replay)	-0.1243	0.0329	78	-3.772	0.0072	-3.0802
(AP10k transfer learning) - (SuperAnimal transfer learning)	-0.0895	0.0329	78	-2.717	0.1330	-2.2185
(AP10k transfer learning) - SuperAnimal zero-shot	0.0508	0.0329	78	1.542	0.7820	1.2594
AP10k zero-shot - (ImageNet transfer learning)	0.3358	0.0329	78	10.197	<.0001	8.3255
AP10k zero-shot - SuperAnimal fine-tune	-0.2306	0.0329	78	-7.001	<.0001	-5.7160
AP10k zero-shot - (SuperAnimal memory replay)	-0.2298	0.0329	78	-6.978	<.0001	-5.6971
AP10k zero-shot - (SuperAnimal transfer learning)	-0.1951	0.0329	78	-5.922	<.0001	-4.8354
AP10k zero-shot - SuperAnimal zero-shot	-0.0548	0.0329	78	-1.663	0.7108	-1.3575
(ImageNet transfer learning) - SuperAnimal fine-tune	-0.5664	0.0329	78	-17.197	<.0001	-14.0415
(ImageNet transfer learning) - (SuperAnimal memory replay)	-0.5657	0.0329	78	-17.174	<.0001	-14.0227
(ImageNet transfer learning) - (SuperAnimal transfer learning)	-0.5309	0.0329	78	-16.119	<.0001	-13.1610
(ImageNet transfer learning) - SuperAnimal zero-shot	-0.3906	0.0329	78	-11.859	<.0001	-9.6830
SuperAnimal fine-tune - (SuperAnimal memory replay)	0.0008	0.0329	78	0.023	1.0000	0.0188
SuperAnimal fine-tune - (SuperAnimal transfer learning)	0.0355	0.0329	78	1.078	0.9595	0.8805
SuperAnimal fine-tune - SuperAnimal zero-shot	0.1758	0.0329	78	5.338	<.0001	4.3585
(SuperAnimal memory replay) - (SuperAnimal transfer learning)	0.0348	0.0329	78	1.055	0.9639	0.8617
(SuperAnimal memory replay) - SuperAnimal zero-shot	0.1751	0.0329	78	5.315	<.0001	4.3397
(SuperAnimal transfer learning) - SuperAnimal zero-shot	0.1403	0.0329	78	4.260	0.0014	3.4780
train data_ratio = 0.5						
AP10k fine-tune - (AP10k transfer learning)	0.0026	0.0329	78	0.079	1.0000	0.0649
AP10k fine-tune - AP10k zero-shot	0.2564	0.0329	78	7.785	<.0001	6.3561
AP10k fine-tune - (ImageNet transfer learning)	0.1495	0.0329	78	4.539	0.0005	3.7058
AP10k fine-tune - SuperAnimal fine-tune	-0.0229	0.0329	78	-0.695	0.9969	-0.5677
AP10k fine-tune - (SuperAnimal memory replay)	-0.0219	0.0329	78	-0.664	0.9977	-0.5419
AP10k fine-tune - (SuperAnimal transfer learning)	-0.0174	0.0329	78	-0.527	0.9995	-0.4307
AP10k fine-tune - SuperAnimal zero-shot	0.2016	0.0329	78	6.122	<.0001	4.9986
(AP10k transfer learning) - AP10k zero-shot	0.2538	0.0329	78	7.705	<.0001	6.2912
(AP10k transfer learning) - (ImageNet transfer learning)	0.1469	0.0329	78	4.459	0.0007	3.6409
(AP10k transfer learning) - SuperAnimal fine-tune	-0.0255	0.0329	78	-0.775	0.9940	-0.6326
(AP10k transfer learning) - (SuperAnimal memory replay)	-0.0245	0.0329	78	-0.743	0.9953	-0.6067
(AP10k transfer learning) - (SuperAnimal transfer learning)	-0.0200	0.0329	78	-0.607	0.9987	-0.4956
(AP10k transfer learning) - SuperAnimal zero-shot	0.1990	0.0329	78	6.043	<.0001	4.9337
AP10k zero-shot - (ImageNet transfer learning)	-0.1069	0.0329	78	-3.246	0.0350	-2.6503
AP10k zero-shot - SuperAnimal fine-tune	-0.2793	0.0329	78	-8.480	<.0001	-6.9238
AP10k zero-shot - (SuperAnimal memory replay)	-0.2783	0.0329	78	-8.448	<.0001	-6.8980
AP10k zero-shot - (SuperAnimal transfer learning)	-0.2738	0.0329	78	-8.312	<.0001	-6.7868
AP10k zero-shot - SuperAnimal zero-shot	-0.0548	0.0329	78	-1.663	0.7108	-1.3575

Table S17. Type-III Analysis of Variance Table for iRodent benchmark mixed model.

	Sum Sq	Mean Sq	NumDF	DenDF	F value	Pr(>F)
cond	1.44	0.21	7.00	78.00	572.88	<.0001
data_ratio	1.08	0.27	4.00	78.00	750.34	<.0001
cond:data_ratio	0.90	0.03	28.00	78.00	89.47	<.0001

(ImageNet transfer learning) - SuperAnimal fine-tune	-0.1724	0.0329	78	-5.234	<.0001	-4.2735
(ImageNet transfer learning) - (SuperAnimal memory replay)	-0.1713	0.0329	78	-5.202	<.0001	-4.2477
(ImageNet transfer learning) - (SuperAnimal transfer learning)	-0.1669	0.0329	78	-5.066	0.0001	-4.1365
(ImageNet transfer learning) - SuperAnimal zero-shot	0.0522	0.0329	78	1.583	0.7586	1.2928
SuperAnimal fine-tune - (SuperAnimal memory replay)	0.0010	0.0329	78	0.032	1.0000	0.0259
SuperAnimal fine-tune - (SuperAnimal transfer learning)	0.0055	0.0329	78	0.168	1.0000	0.1370
SuperAnimal fine-tune - SuperAnimal zero-shot	0.2245	0.0329	78	6.817	<.0001	5.5663
(SuperAnimal memory replay) - (SuperAnimal transfer learning)	0.0045	0.0329	78	0.136	1.0000	0.1112
(SuperAnimal memory replay) - SuperAnimal zero-shot	0.2235	0.0329	78	6.786	<.0001	5.5405
(SuperAnimal transfer learning) - SuperAnimal zero-shot	0.2190	0.0329	78	6.650	<.0001	5.4293
train data_ratio = 1						
AP10k fine-tune - (AP10k transfer learning)	-0.0005	0.0329	78	-0.016	1.0000	-0.0133
AP10k fine-tune - AP10k zero-shot	0.2824	0.0329	78	8.575	<.0001	7.0016
AP10k fine-tune - (ImageNet transfer learning)	0.0346	0.0329	78	1.050	0.9649	0.8570
AP10k fine-tune - SuperAnimal fine-tune	-0.0146	0.0329	78	-0.443	0.9998	-0.3620
AP10k fine-tune - (SuperAnimal memory replay)	-0.0119	0.0329	78	-0.362	1.0000	-0.2956
AP10k fine-tune - (SuperAnimal transfer learning)	-0.0086	0.0329	78	-0.262	1.0000	-0.2141
AP10k fine-tune - SuperAnimal zero-shot	0.2277	0.0329	78	6.913	<.0001	5.6441
(AP10k transfer learning) - AP10k zero-shot	0.2830	0.0329	78	8.591	<.0001	7.0149
(AP10k transfer learning) - (ImageNet transfer learning)	0.0351	0.0329	78	1.066	0.9619	0.8703
(AP10k transfer learning) - SuperAnimal fine-tune	-0.0141	0.0329	78	-0.427	0.9999	-0.3488
(AP10k transfer learning) - (SuperAnimal memory replay)	-0.0114	0.0329	78	-0.346	1.0000	-0.2823
(AP10k transfer learning) - (SuperAnimal transfer learning)	-0.0081	0.0329	78	-0.246	1.0000	-0.2009
(AP10k transfer learning) - SuperAnimal zero-shot	0.2282	0.0329	78	6.929	<.0001	5.6574
AP10k zero-shot - (ImageNet transfer learning)	-0.2479	0.0329	78	-7.526	<.0001	-6.1446
AP10k zero-shot - SuperAnimal fine-tune	-0.2970	0.0329	78	-9.019	<.0001	-7.3636
AP10k zero-shot - (SuperAnimal memory replay)	-0.2944	0.0329	78	-8.937	<.0001	-7.2972
AP10k zero-shot - (SuperAnimal transfer learning)	-0.2911	0.0329	78	-8.837	<.0001	-7.2157
AP10k zero-shot - SuperAnimal zero-shot	-0.0548	0.0329	78	-1.663	0.7108	-1.3575
(ImageNet transfer learning) - SuperAnimal fine-tune	-0.0492	0.0329	78	-1.493	0.8088	-1.2191
(ImageNet transfer learning) - (SuperAnimal memory replay)	-0.0465	0.0329	78	-1.412	0.8492	-1.1526
(ImageNet transfer learning) - (SuperAnimal transfer learning)	-0.0432	0.0329	78	-1.312	0.8917	-1.0712
(ImageNet transfer learning) - SuperAnimal zero-shot	0.1931	0.0329	78	5.863	<.0001	4.7871
SuperAnimal fine-tune - (SuperAnimal memory replay)	0.0027	0.0329	78	0.081	1.0000	0.0665
SuperAnimal fine-tune - (SuperAnimal transfer learning)	0.0060	0.0329	78	0.181	1.0000	0.1479
SuperAnimal fine-tune - SuperAnimal zero-shot	0.2423	0.0329	78	7.356	<.0001	6.0062
(SuperAnimal memory replay) - (SuperAnimal transfer learning)	0.0033	0.0329	78	0.100	1.0000	0.0814
(SuperAnimal memory replay) - SuperAnimal zero-shot	0.2396	0.0329	78	7.275	<.0001	5.9397
(SuperAnimal transfer learning) - SuperAnimal zero-shot	0.2363	0.0329	78	7.175	<.0001	5.8583

Degrees-of-freedom method: kenward-roger

P value adjustment: tukey method for comparing a family of 8 estimates

Table S18. Two-sided pairwise contrasts adjusted with Tukey's method for the iRodent benchmark mixed model.

contrast	estimate	SE	df	t.ratio	p.value	eff.size
train data_ratio = 0.01						
AP10k fine-tune - (AP10k transfer learning)	0.3023	0.0155	78	19.513	<.0001	15.9326
AP10k fine-tune - AP10k zero-shot	0.0275	0.0155	78	1.778	0.6365	1.4517
AP10k fine-tune - (ImageNet transfer learning)	0.4236	0.0155	78	27.339	<.0001	22.3221
AP10k fine-tune - SuperAnimal fine-tune	-0.1605	0.0155	78	-10.359	<.0001	-8.4579
AP10k fine-tune - (SuperAnimal memory replay)	-0.1771	0.0155	78	-11.429	<.0001	-9.3318

AP10k fine-tune - (SuperAnimal transfer learning)	0.2552	0.0155	78	16.469	<.0001	13.4473
AP10k fine-tune - SuperAnimal zero-shot	-0.1541	0.0155	78	-9.947	<.0001	-8.1221
(AP10k transfer learning) - AP10k zero-shot	-0.2748	0.0155	78	-17.735	<.0001	-14.4808
(AP10k transfer learning) - (ImageNet transfer learning)	0.1212	0.0155	78	7.826	<.0001	6.3896
(AP10k transfer learning) - SuperAnimal fine-tune	-0.4628	0.0155	78	-29.872	<.0001	-24.3905
(AP10k transfer learning) - (SuperAnimal memory replay)	-0.4794	0.0155	78	-30.942	<.0001	-25.2644
(AP10k transfer learning) - (SuperAnimal transfer learning)	-0.0472	0.0155	78	-3.044	0.0602	-2.4853
(AP10k transfer learning) - SuperAnimal zero-shot	-0.4565	0.0155	78	-29.461	<.0001	-24.0547
AP10k zero-shot - (ImageNet transfer learning)	0.3960	0.0155	78	25.561	<.0001	20.8704
AP10k zero-shot - SuperAnimal fine-tune	-0.1880	0.0155	78	-12.137	<.0001	-9.9097
AP10k zero-shot - (SuperAnimal memory replay)	-0.2046	0.0155	78	-13.207	<.0001	-10.7836
AP10k zero-shot - (SuperAnimal transfer learning)	0.2276	0.0155	78	14.691	<.0001	11.9956
AP10k zero-shot - SuperAnimal zero-shot	-0.1817	0.0155	78	-11.725	<.0001	-9.5738
(ImageNet transfer learning) - SuperAnimal fine-tune	-0.5841	0.0155	78	-37.698	<.0001	-30.7801
(ImageNet transfer learning) - (SuperAnimal memory replay)	-0.6007	0.0155	78	-38.768	<.0001	-31.6540
(ImageNet transfer learning) - (SuperAnimal transfer learning)	-0.1684	0.0155	78	-10.869	<.0001	-8.8748
(ImageNet transfer learning) - SuperAnimal zero-shot	-0.5777	0.0155	78	-37.286	<.0001	-30.4442
SuperAnimal fine-tune - (SuperAnimal memory replay)	-0.0166	0.0155	78	-1.070	0.9611	-0.8739
SuperAnimal fine-tune - (SuperAnimal transfer learning)	0.4157	0.0155	78	26.828	<.0001	21.9052
SuperAnimal fine-tune - SuperAnimal zero-shot	0.0064	0.0155	78	0.411	0.9999	0.3359
(SuperAnimal memory replay) - (SuperAnimal transfer learning)	0.4323	0.0155	78	27.899	<.0001	22.7791
(SuperAnimal memory replay) - SuperAnimal zero-shot	0.0230	0.0155	78	1.482	0.8148	1.2098
(SuperAnimal transfer learning) - SuperAnimal zero-shot	-0.4093	0.0155	78	-26.417	<.0001	-21.5694
train data_ratio = 0.05						
AP10k fine-tune - (AP10k transfer learning)	0.1026	0.0155	78	6.624	<.0001	5.4083
AP10k fine-tune - AP10k zero-shot	0.0922	0.0155	78	5.948	<.0001	4.8563
AP10k fine-tune - (ImageNet transfer learning)	0.2626	0.0155	78	16.945	<.0001	13.8358
AP10k fine-tune - SuperAnimal fine-tune	-0.1165	0.0155	78	-7.519	<.0001	-6.1394
AP10k fine-tune - (SuperAnimal memory replay)	-0.1367	0.0155	78	-8.823	<.0001	-7.2040
AP10k fine-tune - (SuperAnimal transfer learning)	0.0012	0.0155	78	0.079	1.0000	0.0648
AP10k fine-tune - SuperAnimal zero-shot	-0.0895	0.0155	78	-5.778	<.0001	-4.7175
(AP10k transfer learning) - AP10k zero-shot	-0.0105	0.0155	78	-0.676	0.9974	-0.5520
(AP10k transfer learning) - (ImageNet transfer learning)	0.1599	0.0155	78	10.322	<.0001	8.4275
(AP10k transfer learning) - SuperAnimal fine-tune	-0.2191	0.0155	78	-14.143	<.0001	-11.5477
(AP10k transfer learning) - (SuperAnimal memory replay)	-0.2393	0.0155	78	-15.447	<.0001	-12.6123
(AP10k transfer learning) - (SuperAnimal transfer learning)	-0.1014	0.0155	78	-6.544	<.0001	-5.3435
(AP10k transfer learning) - SuperAnimal zero-shot	-0.1922	0.0155	78	-12.402	<.0001	-10.1258
AP10k zero-shot - (ImageNet transfer learning)	0.1704	0.0155	78	10.998	<.0001	8.9795
AP10k zero-shot - SuperAnimal fine-tune	-0.2087	0.0155	78	-13.467	<.0001	-10.9957
AP10k zero-shot - (SuperAnimal memory replay)	-0.2289	0.0155	78	-14.771	<.0001	-12.0602
AP10k zero-shot - (SuperAnimal transfer learning)	-0.0909	0.0155	78	-5.868	<.0001	-4.7915
AP10k zero-shot - SuperAnimal zero-shot	-0.1817	0.0155	78	-11.725	<.0001	-9.5738
(ImageNet transfer learning) - SuperAnimal fine-tune	-0.3791	0.0155	78	-24.465	<.0001	-19.9752
(ImageNet transfer learning) - (SuperAnimal memory replay)	-0.3993	0.0155	78	-25.768	<.0001	-21.0398
(ImageNet transfer learning) - (SuperAnimal transfer learning)	-0.2613	0.0155	78	-16.866	<.0001	-13.7710
(ImageNet transfer learning) - SuperAnimal zero-shot	-0.3521	0.0155	78	-22.723	<.0001	-18.5533
SuperAnimal fine-tune - (SuperAnimal memory replay)	-0.0202	0.0155	78	-1.304	0.8948	-1.0645
SuperAnimal fine-tune - (SuperAnimal transfer learning)	0.1177	0.0155	78	7.599	<.0001	6.2042
SuperAnimal fine-tune - SuperAnimal zero-shot	0.0270	0.0155	78	1.741	0.6605	1.4219
(SuperAnimal memory replay) - (SuperAnimal transfer learning)	0.1379	0.0155	78	8.902	<.0001	7.2688
(SuperAnimal memory replay) - SuperAnimal zero-shot	0.0472	0.0155	78	3.045	0.0600	2.4864
(SuperAnimal transfer learning) - SuperAnimal zero-shot	-0.0908	0.0155	78	-5.857	<.0001	-4.7823
train data_ratio = 0.1						
AP10k fine-tune - (AP10k transfer learning)	0.0754	0.0155	78	4.867	0.0002	3.9742
AP10k fine-tune - AP10k zero-shot	0.0963	0.0155	78	6.215	<.0001	5.0745
AP10k fine-tune - (ImageNet transfer learning)	0.2229	0.0155	78	14.387	<.0001	11.7470
AP10k fine-tune - SuperAnimal fine-tune	-0.1102	0.0155	78	-7.114	<.0001	-5.8089

AP10k fine-tune - (SuperAnimal memory replay)	-0.1370	0.0155	78	-8.840	<.0001	-7.2178
AP10k fine-tune - (SuperAnimal transfer learning)	-0.0483	0.0155	78	-3.117	0.0497	-2.5447
AP10k fine-tune - SuperAnimal zero-shot	-0.0854	0.0155	78	-5.510	<.0001	-4.4993
(AP10k transfer learning) - AP10k zero-shot	0.0209	0.0155	78	1.348	0.8774	1.1003
(AP10k transfer learning) - (ImageNet transfer learning)	0.1475	0.0155	78	9.520	<.0001	7.7728
(AP10k transfer learning) - SuperAnimal fine-tune	-0.1856	0.0155	78	-11.982	<.0001	-9.7832
(AP10k transfer learning) - (SuperAnimal memory replay)	-0.2124	0.0155	78	-13.707	<.0001	-11.1921
(AP10k transfer learning) - (SuperAnimal transfer learning)	-0.1237	0.0155	78	-7.984	<.0001	-6.5189
(AP10k transfer learning) - SuperAnimal zero-shot	-0.1608	0.0155	78	-10.378	<.0001	-8.4735
AP10k zero-shot - (ImageNet transfer learning)	0.1266	0.0155	78	8.172	<.0001	6.6725
AP10k zero-shot - SuperAnimal fine-tune	-0.2065	0.0155	78	-13.329	<.0001	-10.8834
AP10k zero-shot - (SuperAnimal memory replay)	-0.2333	0.0155	78	-15.055	<.0001	-12.2923
AP10k zero-shot - (SuperAnimal transfer learning)	-0.1446	0.0155	78	-9.332	<.0001	-7.6192
AP10k zero-shot - SuperAnimal zero-shot	-0.1817	0.0155	78	-11.725	<.0001	-9.5738
(ImageNet transfer learning) - SuperAnimal fine-tune	-0.3331	0.0155	78	-21.502	<.0001	-17.5559
(ImageNet transfer learning) - (SuperAnimal memory replay)	-0.3599	0.0155	78	-23.227	<.0001	-18.9648
(ImageNet transfer learning) - (SuperAnimal transfer learning)	-0.2712	0.0155	78	-17.504	<.0001	-14.2917
(ImageNet transfer learning) - SuperAnimal zero-shot	-0.3083	0.0155	78	-19.898	<.0001	-16.2463
SuperAnimal fine-tune - (SuperAnimal memory replay)	-0.0267	0.0155	78	-1.726	0.6708	-1.4089
SuperAnimal fine-tune - (SuperAnimal transfer learning)	0.0619	0.0155	78	3.998	0.0035	3.2642
SuperAnimal fine-tune - SuperAnimal zero-shot	0.0249	0.0155	78	1.604	0.7465	1.3096
(SuperAnimal memory replay) - (SuperAnimal transfer learning)	0.0887	0.0155	78	5.723	<.0001	4.6731
(SuperAnimal memory replay) - SuperAnimal zero-shot	0.0516	0.0155	78	3.329	0.0277	2.7185
(SuperAnimal transfer learning) - SuperAnimal zero-shot	-0.0371	0.0155	78	-2.394	0.2590	-1.9546
train data_ratio = 0.5						
AP10k fine-tune - (AP10k transfer learning)	-0.0659	0.0155	78	-4.253	0.0014	-3.4728
AP10k fine-tune - AP10k zero-shot	0.1747	0.0155	78	11.275	<.0001	9.2056
AP10k fine-tune - (ImageNet transfer learning)	0.0735	0.0155	78	4.743	0.0002	3.8730
AP10k fine-tune - SuperAnimal fine-tune	-0.1217	0.0155	78	-7.855	<.0001	-6.4133
AP10k fine-tune - (SuperAnimal memory replay)	-0.1140	0.0155	78	-7.361	<.0001	-6.0101
AP10k fine-tune - (SuperAnimal transfer learning)	-0.1196	0.0155	78	-7.720	<.0001	-6.3030
AP10k fine-tune - SuperAnimal zero-shot	-0.0070	0.0155	78	-0.451	0.9998	-0.3682
(AP10k transfer learning) - AP10k zero-shot	0.2406	0.0155	78	15.528	<.0001	12.6784
(AP10k transfer learning) - (ImageNet transfer learning)	0.1394	0.0155	78	8.997	<.0001	7.3458
(AP10k transfer learning) - SuperAnimal fine-tune	-0.0558	0.0155	78	-3.601	0.0124	-2.9405
(AP10k transfer learning) - (SuperAnimal memory replay)	-0.0481	0.0155	78	-3.108	0.0510	-2.5373
(AP10k transfer learning) - (SuperAnimal transfer learning)	-0.0537	0.0155	78	-3.466	0.0186	-2.8301
(AP10k transfer learning) - SuperAnimal zero-shot	0.0589	0.0155	78	3.802	0.0066	3.1046
AP10k zero-shot - (ImageNet transfer learning)	-0.1012	0.0155	78	-6.531	<.0001	-5.3327
AP10k zero-shot - SuperAnimal fine-tune	-0.2964	0.0155	78	-19.129	<.0001	-15.6189
AP10k zero-shot - (SuperAnimal memory replay)	-0.2887	0.0155	78	-18.635	<.0001	-15.2157
AP10k zero-shot - (SuperAnimal transfer learning)	-0.2943	0.0155	78	-18.994	<.0001	-15.5086
AP10k zero-shot - SuperAnimal zero-shot	-0.1817	0.0155	78	-11.725	<.0001	-9.5738
(ImageNet transfer learning) - SuperAnimal fine-tune	-0.1952	0.0155	78	-12.598	<.0001	-10.2863
(ImageNet transfer learning) - (SuperAnimal memory replay)	-0.1875	0.0155	78	-12.104	<.0001	-9.8831
(ImageNet transfer learning) - (SuperAnimal transfer learning)	-0.1931	0.0155	78	-12.463	<.0001	-10.1759
(ImageNet transfer learning) - SuperAnimal zero-shot	-0.0805	0.0155	78	-5.194	<.0001	-4.2411
SuperAnimal fine-tune - (SuperAnimal memory replay)	0.0077	0.0155	78	0.494	0.9997	0.4032
SuperAnimal fine-tune - (SuperAnimal transfer learning)	0.0021	0.0155	78	0.135	1.0000	0.1104
SuperAnimal fine-tune - SuperAnimal zero-shot	0.1147	0.0155	78	7.404	<.0001	6.0451
(SuperAnimal memory replay) - (SuperAnimal transfer learning)	-0.0056	0.0155	78	-0.359	1.0000	-0.2928
(SuperAnimal memory replay) - SuperAnimal zero-shot	0.1071	0.0155	78	6.910	<.0001	5.6419
(SuperAnimal transfer learning) - SuperAnimal zero-shot	0.1126	0.0155	78	7.269	<.0001	5.9348
train data_ratio = 1						
AP10k fine-tune - (AP10k transfer learning)	-0.0928	0.0155	78	-5.990	<.0001	-4.8906
AP10k fine-tune - AP10k zero-shot	0.2125	0.0155	78	13.712	<.0001	11.1957
AP10k fine-tune - (ImageNet transfer learning)	0.0278	0.0155	78	1.793	0.6266	1.4640

AP10k fine-tune - SuperAnimal fine-tune	-0.1061	0.0155	78	-6.849	<.0001	-5.5924
AP10k fine-tune - (SuperAnimal memory replay)	-0.1134	0.0155	78	-7.317	<.0001	-5.9741
AP10k fine-tune - (SuperAnimal transfer learning)	-0.1041	0.0155	78	-6.720	<.0001	-5.4868
AP10k fine-tune - SuperAnimal zero-shot	0.0308	0.0155	78	1.986	0.4975	1.6219
(AP10k transfer learning) - AP10k zero-shot	0.3053	0.0155	78	19.702	<.0001	16.0863
(AP10k transfer learning) - (ImageNet transfer learning)	0.1206	0.0155	78	7.783	<.0001	6.3546
(AP10k transfer learning) - SuperAnimal fine-tune	-0.0133	0.0155	78	-0.860	0.9887	-0.7019
(AP10k transfer learning) - (SuperAnimal memory replay)	-0.0206	0.0155	78	-1.327	0.8857	-1.0835
(AP10k transfer learning) - (SuperAnimal transfer learning)	-0.0113	0.0155	78	-0.730	0.9958	-0.5963
(AP10k transfer learning) - SuperAnimal zero-shot	0.1236	0.0155	78	7.976	<.0001	6.5125
AP10k zero-shot - (ImageNet transfer learning)	-0.1847	0.0155	78	-11.919	<.0001	-9.7317
AP10k zero-shot - SuperAnimal fine-tune	-0.3186	0.0155	78	-20.561	<.0001	-16.7881
AP10k zero-shot - (SuperAnimal memory replay)	-0.3258	0.0155	78	-21.029	<.0001	-17.1698
AP10k zero-shot - (SuperAnimal transfer learning)	-0.3166	0.0155	78	-20.432	<.0001	-16.6825
AP10k zero-shot - SuperAnimal zero-shot	-0.1817	0.0155	78	-11.725	<.0001	-9.5738
(ImageNet transfer learning) - SuperAnimal fine-tune	-0.1339	0.0155	78	-8.642	<.0001	-7.0564
(ImageNet transfer learning) - (SuperAnimal memory replay)	-0.1411	0.0155	78	-9.110	<.0001	-7.4381
(ImageNet transfer learning) - (SuperAnimal transfer learning)	-0.1319	0.0155	78	-8.513	<.0001	-6.9508
(ImageNet transfer learning) - SuperAnimal zero-shot	0.0030	0.0155	78	0.193	1.0000	0.1579
SuperAnimal fine-tune - (SuperAnimal memory replay)	-0.0072	0.0155	78	-0.467	0.9998	-0.3817
SuperAnimal fine-tune - (SuperAnimal transfer learning)	0.0020	0.0155	78	0.129	1.0000	0.1056
SuperAnimal fine-tune - SuperAnimal zero-shot	0.1369	0.0155	78	8.836	<.0001	7.2143
(SuperAnimal memory replay) - (SuperAnimal transfer learning)	0.0092	0.0155	78	0.597	0.9988	0.4873
(SuperAnimal memory replay) - SuperAnimal zero-shot	0.1441	0.0155	78	9.303	<.0001	7.5960
(SuperAnimal transfer learning) - SuperAnimal zero-shot	0.1349	0.0155	78	8.706	<.0001	7.1087

Degrees-of-freedom method: kenward-roger

P value adjustment: tukey method for comparing a family of 8 estimates

Table S19. Spatial Pyramid. Exact two-sample one-sided Kolmogorov-Smirnov test.

	D	*p*
Smear Lab mouse	1	<.0001
ood_ Mathis MausHaus	0.33	.27
Golden Lab Mouse	0.75	<.0001

Table S20. Type-III Analysis of Variance Table for the mixed model relative to the quantification of video adaptation in terms of keypoint jittering.

	Sum Sq	Mean Sq	NumDF	DenDF	F value	Pr(>F)
video	807.99	134.67	6.00	2.00	2.94	0.2753
cond	8699.68	8699.68	1.00	23286.00	190.03	<.0001
video:cond	11630.29	1938.38	6.00	23286.00	42.34	<.0001

Table S21. Two-sided pairwise contrasts adjusted with Tukey's method for the mixed model relative to the quantification of video adaptation in terms of keypoint jittering.

contrast	estimate	SE	df	z.ratio	p.value	eff.size
video = DLC-Openfield						
after_adapt - before_adapt	-6.3923	0.3791	Inf	-16.860	<.0001	-0.9447
video = Dog						
after_adapt - before_adapt	-0.1828	0.1983	Inf	-0.922	0.3566	-0.0270
video = Elk						
after_adapt - before_adapt	-1.6065	0.1674	Inf	-9.596	<.0001	-0.2374
video = Golden Lab						
after_adapt - before_adapt	-0.3877	0.7839	Inf	-0.495	0.6209	-0.0573
video = Horse						
after_adapt - before_adapt	-6.6062	0.7974	Inf	-8.285	<.0001	-0.9764
video = MausHaus						
after_adapt - before_adapt	-1.5839	0.5878	Inf	-2.695	0.0070	-0.2341
video = Smear Lab						
after_adapt - before_adapt	-1.8673	0.1373	Inf	-13.603	<.0001	-0.2760
Degrees-of-freedom method: asymptotic						

Table S22. One-way repeated measures ANOVA table testing for differences in adaptation gain between smoothing methods.

	Source	ddof1	ddof2	F	p-unc	p-GG-corr	ng2	eps	sphericity	W-spher	p-spher
0	method	2	58	25.078469	0.000000	0.000018	0.363796	0.520776	False	0.079789	0.000000

Table S23. Post-hoc pairwise contrasts for the adaptation gain ANOVA.

A	B	T	dof	alternative	p-unc	cohen
Kalman filter	Self-pacing	-4.725	29.000	two-sided	<.0001	-1.226
Kalman filter	Video adaptation	-5.319	29.000	two-sided	<.0001	-1.358
Self-pacing	Video adaptation	-3.261	29.000	two-sided	0.003	-0.785

Table S24. Paired t-test testing for differences in robustness gain between self-pacing and video adaptation.

	T	dof	alternative	p-val	CI95%	cohen-d
T-test	-15.473	29	two-sided	0.000	[-4.36 -3.34]	3.124

Table S25. Type-III Analysis of Variance Table for OFT linear mixed effect model.

	Sum Sq	Mean Sq	NumDF	DenDF	F value	Pr(>F)
method	0.1371	0.0457	3	453	0.9988	0.3932
action	12.1056	12.1056	1	453	264.6151	<0.0001
method:action	0.0202	0.0067	3	453	0.1474	0.9313

Table S26. MABe Results with SA-TVM zero-shot vs. Official MABe pose data. We show that with SuperAnimal keypoints, we get same performance (independent t-test; t=-.02, p=.99) in downstream action segmentation as the official pose does in all 13 considered tasks (1), even though our model is never trained on MABe videos. This demonstrates the effectiveness of our models in downstream action segmentation tasks. To qualitatively support our results see Supplementary Video 6.

Task No.	Official MABe pose	SuperAnimal zero-shot
T0	0.095018	0.095018
T1	0.096345	0.096350
T2	0.657165	0.657245
T3	0.020959	0.020963
T4	0.34015	0.34020
T5	0.718520	0.718519
T6	0.565967	0.565954
T7	0.261730	0.261697
T8	0.005427	0.005427
T9	0.025384	0.025381
T10	0.021717	0.021703
T11	0.107985	0.107988
T12	0.610986	0.610956

Supplementary Notes

Model Cards

We provide Model Cards for the two major outputs, SA-TVM and SA-Q. These are also available on HuggingFace with the model weights, at https://huggingface.co/mwmathis/DeepLabCutModelZoo-SuperAnimal-Quadruped and https://huggingface.co/mwmathis/DeepLabCutModelZoo-SuperAnimal-TopViewMouse

Model Card: SuperAnimal-TopViewMouse (DLCRNet backbone and/or HRNet-w32)

Model Details

- SuperAnimal-TopviewMouse model developed by the Mathis Lab in 2023, and trained to predict mouse 27 key points from a given top view image.
- DLCRNet (2) or HRNet-w32 was trained on the TopviewMouse-5K dataset.
- Models were trained within the DeepLabCut framework or mmpose (HRNet-w32). You can use this model simply with our light-weight loading package called DLCLibrary. Here is an example usage:

```
from pathlib import Path
from dlclibrary import download_huggingface_model
# Creates a folder and downloads the model to it
model_dir = Path("./superanimal-topviewmouse_model_dlcrnet")
model_dir.mkdir()
download_huggingface_model("superanimal_topviewmouse_dlcrnet", model_dir)
```

Intended Use

- Intended to be used for pose tracking of lab mice videos filmed from an overhead view. The models can be used as a plug-and-play solution if extremely high precision is not required (we benchmark the zero-shot performance in the paper). Otherwise, it is recommended to also be used as the weights for transfer learning and fine-tuning.
- Intended for academic and research professionals working in fields related to animal behavior, neuroscience, biomechanics, and ecology.
- Not suitable for other species and other camera views. Also not suitable for videos that look dramatically different from those we show in the paper.

Factors

- Based on the known robustness issues of neural networks, the relevant factors include the lighting, contrast and resolution of the video frames. The presence of objects might also cause false detections of the mice and keypoints. When two or more animals are very close, it could cause the top-down detectors to only detect one animal, if used without further fine-tuning.

Metrics

- Mean Average Precision (mAP)
- Root Mean Square Error (RMSE)

Evaluation Data

- The test split of TopViewMouse-5K and in the paper on two benchmarks, DLC Openfield and TriMouse.

Training Data

- **3CSI, BM, EPM, LDB, OFT** See full details at (3) and (4).
- **BlackMice** See full details at (5).
- **WhiteMice** Courtesy of Prof. Sam Golden and Nastacia Goodwin. See details in SIMBA (6).
- **TriMouse** See full details at (2).
- **DLC-Openfield** See full details at (7).
- **Kiehn-Lab-Openfield, Swimming, and Treadmill** Courtesy of Prof. Ole Kiehn, Dr. Jared Cregg, and Prof. Carmelo Bellardita; see details at (8).
- **MausHaus** We collected video data from five single-housed C57BL/6J male and female mice in an extended home cage, carried out in the laboratory of Mackenzie Mathis at Harvard University and also EPFL (temperature of housing was 20-25C, humidity 20-50%). Data were recorded at 30Hz with 640 × 480 pixels resolution acquired with White Matter, LLC eV cameras. Annotators localized 26 keypoints across 322 frames sampled from within DeepLabCut using the k-means clustering approach (9). All experimental procedures for mice were in accordance with the National Institutes of Health Guide for the Care and Use of Laboratory Animals and approved by the Harvard Institutional Animal Care and Use Committee (IACUC) (n=1 mouse), and by the Veterinary Office of the Canton of Geneva (Switzerland; license GE01) (n=4 mice). MausHaus data is banked at https://zenodo.org/records/10593101.

Ethical Considerations

- Data was collected with IUCAC or other governmental approval. Each individual dataset used in training reports the ethics approval they obtained.

Caveats and Recommendations

- The model may have reduced accuracy in scenarios with extremely varied lighting conditions or atypical mouse characteristics not well-represented in the training data. For example, this dataset only has one set of white mice, therefore it may not generalize well to diverse settings of white lab mice.
- Please note that each training dataset was labeled by separate labs and different individuals, therefore while we map names to a unified pose vocabulary, there will be annotator bias in keypoint placement (See our Supplementary Note on annotator bias).
- Note the dataset is primarily using C56Blk6/J mice and only some CD1 examples.
- We recommend if performance is not as good as you need it to be, first try our video adaptation, or fine-tune these weights with your own labeling.

License

- This software may not be used to harm any animal deliberately. Released under a modified MIT license. Please see details at `https://huggingface.co/mwmathis/DeepLabCutModelZoo-SuperAnimal-TopViewMouse`.

Quantitative Analyses

- See details at in Figure 1.

Model Card: SuperAnimal-Quadruped (HRNetw32)

Model Details

- SuperAnimal-Quadruped model developed by the Mathis Lab in 2023, trained to predict quadruped pose from images.
- The main backbone model is an HRNet-w32 (10) trained on our Quadruped-80K dataset.
- We also release a top-down detector trained on the same data with Faster R-CNN (11).
- You can use this model simply with our light-weight loading package called DLCLibrary. Here is an example usage:

```
from pathlib import Path
from dlclibrary import download_huggingface_model
# Creates a folder and downloads the model to it
model_dir = Path("./superanimal_quadruped_hrnetw32")
model_dir.mkdir()
download_huggingface_model("superanimal_hrnetw32", model_dir)

```

Intended Use

- Intended to be used for pose estimation of quadruped images taken from side-view. The model serves a better starting point than ImageNet weights in downstream datasets such as AP-10K.
- Intended for academic and research professionals working in fields related to animal behavior, such as neuroscience and ecology.
- Not suitable as a zeros-shot model for applications that require high keypiont precision, but can be fine-tuned with minimal data to reach human-level accuracy. Also not suitable for videos that look dramatically different from those we show in the paper.

Factors

- Based on the known robustness issues of neural networks, the relevant factors include the lighting, contrast and resolution of the video frames. The present of objects might also cause false detections and erroneous keypoints. When two or more animals are extremely close, it could cause the top-down detectors to only detect only one animal, if used without further fine-tuning or with a method such as BUCTD (12).

Metrics

- Mean Average Precision (mAP)
- Root Mean Square Error (RMSE)
- Normalized Error (NE)

Evaluation Data

- In the paper we benchmark on AP-10K, AnimalPose, Horse-10, and iRodent using a leave-one-out strategy. Here, we provide the model that has been trained on all datasets (see below), therefore it should be considered “fine-tuned" on all animal training data listed below. This model is meant for production and evaluation in downstream scientific applications.

Training Data

- **AwA-Pose** Quadruped dataset, see full details at (13).
- **AnimalPose** See full details at (14).
- **AcinoSet** See full details at (15).
- **Horse-30** Horse-30 dataset, benchmark task is called Horse-10; See full details at (16).
- **StanfordDogs** See full details at (17, 18).
- **AP-10K** See full details at (19).
- **APT-36K** See full details at (20)
- **iRodent** We utilized the iNaturalist API functions for scraping observations with the taxon ID of Suborder Myomorpha (21). The functions allowed us to filter the large amount of observations down to the ones with photos under the CC BY-NC creative license. The most common types of rodents from the collected observations are Muskrat (Ondatra zibethicus), Brown Rat (Rattus norvegicus), House Mouse (Mus musculus), Black Rat (Rattus rattus), Hispid Cotton Rat (Sigmodon hispidus), Meadow Vole (Microtus pennsylvanicus), Bank Vole (Clethrionomys glareolus), Deer Mouse (Peromyscus maniculatus), White-footed Mouse (Peromyscus leucopus), Striped Field Mouse (Apodemus agrarius). We then generated segmentation masks over target animals in the data by processing the media through an algorithm we designed that uses a Mask Region Based Convolutional Neural Networks(Mask R-CNN) (22) model with a ResNet-50-FPN backbone (23), pretrained on the COCO datasets (24). The processed 443 images were then manually labeled with pose annotations, and bounding boxes were generated by running Mega Detector (25) on the images, which were then manually verified. iRodent data is banked at `https://zenodo.org/record/8250392`.

  An image with the keypoint guide can be found in Supplementary Figure S1.

Ethical Considerations

- No experimental data was collected for this model; all datasets used are cited.

Caveats and Recommendations

- The model may have reduced accuracy in scenarios with extremely varied lighting conditions or atypical animal characteristics not well-represented in the training data.
- Please note that each dataset was labeled by separate labs and separate individuals, therefore while we map names to a unified pose vocabulary (found here: https://github.com/AdaptiveMotorControlLab/modelzoo-figures), there will be annotator bias in keypoint placement (See our Supplementary Note on annotator bias).
- Note the dataset is highly diverse across species, but collectively has more representation of domesticated animals like dogs, cats, horses, and cattle.
- We recommend if performance is not as good as you need it to be, first try video adaptation (see Ye et al. 2023), or fine-tune these weights with your own labeling.

License

- This software may not be used to harm any animal deliberately. Released under a modified MIT license. Please see details at `https://huggingface.co/mwmathis/DeepLabCutModelZoo-SuperAnimal-Quadruped`.

Quantitative Analyses

- See details at in Figure 2.

Datasheet: TopViewMouse-5K dataset

Motivation

For what purpose was the dataset created? Was there a specific task in mind? Was there a specific gap that needed to be filled? Please provide a description.

We collected publicly available datasets from the community and additionally contribute MausHaus dataset. The purpose is to provide the community a unified vocabulary dataset for training pose models, and to help the community reproduce our findings. This dataset is used to train models with the SuperAnimal method for mouse top-view pose estimation. The dataset was created intentionally with that task in mind, focusing on covering diverse lab settings of mice.

Who created this dataset (e.g., which team, research group) and on behalf of which entity (e.g., company, institution, organization)?

The merged dataset was created by Shaokai Ye, Ph.D. student at The Mathis Lab of Adaptive Intelligence, EPFL and checked by all co-authors. The merged dataset includes the following:

1. 3CSI, BM, EPM, LDB, OFT datasets, from the lab of Prof. Johannes Bohacek; see details at (3) and (4).
2. BlackMice, from the lab of Prof. Chang; see details at (5).
3. WhiteMice, courtesy of Prof. Sam Golden and Nastacia Goodwin; see details in SIMBA (6).
4. TriMouse benchmark dataset, see details at (2).
5. DLC-Openfield, see details at (7).
6. Kiehn-Lab-Openfield, Swimming, and Treadmill, courtesy of Prof. Ole Kiehn, Dr. Jared Cregg, and Prof. Carmelo Bellardita; see details at (8).
7. MausHaus dataset, collected in the lab of Prof. Mackenzie Mathis at Harvard University and EPFL (26).

Who funded the creation of the dataset? If there is an associated grant, please provide the name of the grantor and the grant name and number.

Each individual paper denotes the funding for the work, therefore check the references. For the newly created MausHaus data, it was funded by start-up funds to Prof. Mackenzie Mathis at the Rowland Institute of Harvard and at EPFL.

Any other comments?

None.

Composition

What do the instances that comprise the dataset represent (e.g., documents, photos, people, countries)? Are there multiple types of instances (e.g., movies, users, and ratings; people and interactions between them; nodes and edges)? Please provide a description.

The instances are images of mice extracted from the top-view video coupled with the human annotated keypoints. Videos have different resolutions, number of animals per frame, number of annotated keypoints as well as frame frequencies. To our best knowledge, frames were only annotated once per instance.

How many instances are there in total (of each type, if appropriate)?

The merged dataset consists of approximately 5,000 frames. For more information see Supplementary Figure S1.

Does the dataset contain all possible instances or is it a sample (not necessarily random) of instances from a larger set? If the dataset is a sample, then what is the larger set? Is the sample representative of the larger set (e.g., geographic coverage)? If so, please describe how this representativeness was validated/verified. If it is not representative of the larger set, please describe why not (e.g., to cover a more diverse range of instances, because instances were withheld or unavailable).

The merged dataset contains all possible instances from each individual source. For MausHaus, the frames were extracted from multiple different mice and videos using kmeans clustering then labeled within the DeepLabCut software package (versions 2.0.7-2.2 were used).

What data does each instance consist of? "Raw" data (e.g., unprocessed text or images) or features? In either case, please provide a description.

Each instance in the dataset comprises a top-view image featuring one or more mice. Accompanying these images are human annotated keypoints for each individual mouse, which detail specific points of interest or markers on the animal's body. These keypoints provide valuable information for pose estimation and behavioral analysis.

Is there a label or target associated with each instance? If so, please provide a description.

The labels are the 2D coordinates (x, y in pixel space) and visibility flags (undefined, unlabeled if occluded, labeled) per each keypoint for each dataset.

Is any information missing from individual instances? If so, please provide a description, explaining why this information is missing (e.g., because it was unavailable). This does not include intentionally removed information, but might include, e.g., redacted text.

Unknown to the authors of the merged dataset.

Are relationships between individual instances made explicit (e.g., users' movie ratings, social network links)? If so, please describe how these relationships are made explicit.

In the dataset of frames extracted from top-view videos of mice, the relationships between individual instances (frames) are not explicitly defined in terms of behavioral interactions or social links. Instead, the dataset primarily focuses on isolated frames as individual instances. Any temporal or behavioral relationships between the frames would be implicit, derived from the sequence in which they appear in the videos.

Are there recommended data splits (e.g., training, development/validation, testing)? If so, please provide a description of these splits, explaining the rationale behind them.

The dataset is partitioned into a train-test split with a ratio of 95:5. This distribution is established to rigorously evaluate the model's efficacy on a set of data distinct from those used during its training phase.

Are there any errors, sources of noise, or redundancies in the dataset? If so, please provide a description.

There are two primary sources of errors in our dataset: firstly, annotation errors from the annotators of individual datasets may exist - we did not correct any original data source; and secondly, imperfections in the projection of keypoints from the original keypoint space to the superset keypoint space cannot be guaranteed to not have occurred, although the authors did their best efforts to avoid such errors. Please see the pre-processing Methods section for more details and for the conversion table that the authors created.

Is the dataset self-contained, or does it link to or otherwise rely on external resources (e.g., websites, tweets, other datasets)? If it links to or relies on external resources, a) are there guarantees that they will exist, and remain constant, over time; b) are there official archival versions of the complete dataset (i.e., including the external resources as they existed at the time the dataset was created); c) are there any restrictions (e.g., licenses, fees) associated with any of the external resources that might apply to a future user? Please provide descriptions of all external resources and any restrictions associated with them, as well as links or other access points, as appropriate.

The merged single source dataset is self-contained and does not rely on external link that might change over time. Individual dataset links could be modified.

Does the dataset contain data that might be considered confidential (e.g., data that is protected by legal privilege or by doctor-patient confidentiality, data that includes the content of individuals non-public communications)? If so, please provide a description.

To our best knowledge, no such data is included, and all data was collected under ethics approval for animal research.

Does the dataset contain data that, if viewed directly, might be offensive, insulting, threatening, or might otherwise cause anxiety? If so, please describe why.

Unknown to the authors of the datasheet, but the images are of uninjured animals in freely moving settings in laboratories, therefore we do not anticipate they cause alarm for humans.

Does the dataset relate to people? If not, you may skip the remaining questions in this section.

No

Any other comments?

None.

Collection Process

How was the data associated with each instance acquired? Was the data directly observable (e.g., raw text, movie ratings), reported by subjects (e.g., survey responses), or indirectly inferred/derived from other data (e.g., part-of-speech tags, model-based guesses for age or language)? If data was reported by subjects or indirectly inferred/derived from other data, was the data validated/verified? If so, please describe how.

Individual datasets before merging were acquired from published papers or annotated by authors of the paper.
Datasets are validated and verified by the original dataset creators and later verified by authors of this paper.

What mechanisms or procedures were used to collect the data (e.g., hardware apparatus or sensor, manual human curation, software program, software API)? How were these mechanisms or procedures validated?

For MausHaus dataset we collected video data from five single-housed C57BL/6J male and female mice in an extended home cage, carried out in the laboratory of Mackenzie Mathis at Harvard University and also EPFL. Data were recorded with White Matter, LLC eV cameras. Annotators localized 27 keypoints across 322 frames sampled from within DeepLabCut using the k-means clustering approach (9). All experimental procedures for mice were in accordance with the National Institutes of Health Guide for the Care and Use of Laboratory Animals and approved by the Harvard Institutional Animal Care and Use Committee (IACUC) (n=1 mouse), and by the Veterinary Office of the Canton of Geneva (Switzerland; license GE01) (n=4 mice).

If the dataset is a sample from a larger set, what was the sampling strategy (e.g., deterministic, probabilistic with specific sampling probabilities)?

For publicly available data, please see their methods. For MausHaus, it was sampled via k-means clustering of videos.

Who was involved in the data collection process (e.g., students, crowdworkers, contractors) and how were they compensated (e.g., how much were crowdworkers paid)?

We do not have information on the publicly available datasets. MausHaus was annotated by Prof. Mackenzie Mathis as part of her employment at either Harvard University or EPFL.

Over what time frame was the data collected? Does this time frame match the creation time frame of the

data associated with the instances (e.g., recent crawl of old news articles)? If not, please describe the time frame in which the data associated with the instances was created.
Data was collected from 2019-2023.

Were any ethical review processes conducted (e.g., by an institutional review board)? If so, please provide a description of these review processes, including the outcomes, as well as a link or other access point to any supporting documentation.
Yes, every individual paper we sourced data from included a relevant ethical approval to collect data from mice.

Does the dataset relate to people? If not, you may skip the remaining questions in this section.
No.

Preprocessing/cleaning/labeling

Was any preprocessing/cleaning/labeling of the data done (e.g., discretization or bucketing, tokenization, part-of-speech tagging, SIFT feature extraction, removal of instances, processing of missing values)? If so, please provide a description. If not, you may skip the remainder of the questions in this section.
Data came from multiple sources and in multiple formats. To homogenize different annotation formats (COCO-style, DeepLabCut format, etc.), we implemented a generalized data converter. We parsed public datasets and reformatted them into a single project (either DeepLabCut format or COCO). Besides data conversion, the generalized data converter also implements key steps for the panoptic animal pose estimation task formulation, but no individual keypoints were changed.

Was the "raw" data saved in addition to the preprocessed/cleaned/labeled data (e.g., to support unanticipated future uses)? If so, please provide a link or other access point to the "raw" data.
The raw data was used, and can be extracted from the corresponding original source.

Is the software used to preprocess/clean/label the instances available? If so, please provide a link or other access point. Yes, the conversion table is available at https://github.com/AdaptiveMotorControlLab/modelzoo-figures.

Any other comments?
None.

Uses

Has the dataset been used for any tasks already? If so, please provide a description.
At the time of publication, only the original paper has used the dataset.

Is there a repository that links to any or all papers or systems that use the dataset? If so, please provide a link or other access point.
We suggest to check the citations of original paper sources.

What (other) tasks could the dataset be used for?
The dataset could be used for anything related to mouse pose estimation.

Is there anything about the composition of the dataset or the way it was collected and preprocessed/cleaned/labeled that might impact future uses? For example, is there anything that a future user might need to know to avoid uses that could result in unfair treatment of individuals or groups (e.g., stereotyping, quality of service issues) or other undesirable harms (e.g., financial harms, legal risks) If so, please provide a description. Is there anything a future user could do to mitigate these undesirable harms?
Keypoint annotations from individual datasets were projected to a super-set keypoint space which represents this dataset. The model that is trained on this dataset might have a bias on keypoints that are more common in the individual datasets and might have larger errors on keypoints that are under-represented in the source datasets.

Are there tasks for which the dataset should not be used? If so, please provide a description.
The dataset cannot be used to harm any animal. The dataset should not be used to train a model that is expected to be directly used (i.e., without further fine-tuning) for applications that require extremely high-precision, as there were annotator bias from the source datasets.

Any other comments?
None.

Distribution

Will the dataset be distributed to third parties outside of the entity (e.g., company, institution, organization) on behalf of which the dataset was created? If so, please provide a description.
Yes, the dataset will be publicly available with a license for use.

How will the dataset will be distributed (e.g., tarball on website, API, GitHub) Does the dataset have a digital object identifier (DOI)?
The dataset is distributed on zenodo (27).

When will the dataset be distributed?
The merged dataset is released with this this paper).

Will the dataset be distributed under a copyright or other intellectual property (IP) license, and/or under applicable terms of use (ToU)? If so, please describe this license and/or ToU, and provide a link or other access point to, or otherwise reproduce, any relevant licensing terms or ToU, as well as any fees associated with these restrictions.
The data copyright belongs to the authors of the original datasets.

Have any third parties imposed IP-based or other restrictions on the data associated with the instances? If so, please describe these restrictions, and provide a link or other access point to, or otherwise reproduce, any relevant licensing terms, as well as any fees associated with these restrictions.

Yes, please check the original sources. We assume no liability or guarantees on this model's use.

Do any export controls or other regulatory restrictions apply to the dataset or to individual instances? If so, please describe these restrictions, and provide a link or other access point to, or otherwise reproduce, any supporting documentation.

Unknown.

Any other comments?

None.

Maintenance

Who will be supporting/hosting/maintaining the dataset?

The dataset will be hosted on zenodo.

How can the owner/curator/manager of the dataset be contacted (e.g., email address)?

The head of The Mathis Lab of Adaptive Intelligence, Mackenzie Mathis, can be contacted at mackenzie.mathis@epfl.ch.

Is there an erratum? If so, please provide a link or other access point.

None.

Will the dataset be updated (e.g., to correct labeling errors, add new instances, delete instances)? If so, please describe how often, by whom, and how updates will be communicated to users (e.g., mailing list, GitHub)?

Not at this time.

If the dataset relates to people, are there applicable limits on the retention of the data associated with the instances (e.g., were individuals in question told that their data would be retained for a fixed period of time and then deleted)? If so, please describe these limits and explain how they will be enforced.

No.

Will older versions of the dataset continue to be supported/hosted/maintained? If so, please describe how. If not, please describe how its obsolescence will be communicated to users.

The data is banked on zenodo.

If others want to extend/augment/build on/contribute to the dataset, is there a mechanism for them to do so? If so, please provide a description. Will these contributions be validated/verified? If so, please describe how. If not, why not? Is there a process for communicating/distributing these contributions to other users? If so, please provide a description.

Others may do so and should contact the original authors about incorporating fixes/extensions.

Any other comments?

None.

Datasheet: Quadruped-80K dataset

Motivation

For what purpose was the dataset created? Was there a specific task in mind? Was there a specific gap that needed to be filled? Please provide a description.

We collected publicly available datasets from the community and additionally contribute iRodent dataset. The purpose is to provide the community with a unified vocabulary dataset for training pose models, and to help the community reproduce our findings. This dataset is used to train models with the SuperAnimal method for quadruped pose estimation. The dataset was created intentionally with that task in mind, focusing on covering animals in the wild.

Who created this dataset (e.g., which team, research group) and on behalf of which entity (e.g., company, institution, organization)?

The merged dataset was created by Shaokai Ye, Ph.D. student at The Mathis Lab of Adaptive Intelligence, EPFL and checked by all co-authors. The merged dataset includes the following:

1. **AwA-Pose** Quadruped dataset, see full details at (13).
2. **AnimalPose** See full details at (14).
3. **AcinoSet** See full details at (15).
4. **Horse-30** Horse-30 dataset, benchmark task is called Horse-10; See full details at (16).
5. **StanfordDogs** See full details at (17, 18).
6. **AP-10K** See full details at (19).
7. **APT-36K** See full details at (20)
8. **iRodent** We utilized the iNaturalist API functions for scraping observations with the taxon ID of Suborder Myomorpha (21). The functions allowed us to filter the large amount of observations down to the ones with photos under the CC BY-NC creative license. The most common types of rodents from the collected observations are Muskrat (Ondatra zibethicus), Brown Rat (Rattus norvegicus), House Mouse (Mus musculus), Black Rat (Rattus rattus), Hispid Cotton Rat (Sigmodon hispidus), Meadow Vole (Microtus pennsylvanicus), Bank Vole (Clethrionomys glareolus), Deer Mouse (Peromyscus maniculatus), White-footed Mouse (Peromyscus leucopus), Striped Field Mouse (Apodemus agrarius). We then generated segmentation masks over target animals in the data by processing the media through an algorithm we designed that uses a Mask Region Based Convolutional Neural Networks(Mask R-CNN) (22) model with a ResNet-50-FPN backbone (23), pretrained on the COCO datasets (24). The processed 443 images were then manually labeled with pose annotations, and bounding boxes were generated by running Mega Detector (25) on the images, which were then manually verified. iRodent data is banked at `https://zenodo.org/record/8250392`.

Who funded the creation of the dataset? If there is an associated grant, please provide the name of the grantor and the grant name and number.

Each individual paper denotes the funding for the work, therefore check the references. For the newly created iRodent data, it was funded by start-up funds to Prof. Mackenzie Mathis at EPFL.

Any other comments?

None.

Composition

What do the instances that comprise the dataset represent (e.g., documents, photos, people, countries)? Are there multiple types of instances (e.g., movies, users, and ratings; people and interactions between them; nodes and edges)? Please provide a description.

The instances are images of animals extracted from the side-view images coupled with the human annotated keypoints. Videos/images have different resolutions, number of animals per frame, number of annotated keypoints as well as frame frequencies. To our best knowledge, frames were only annotated once per instance.

How many instances are there in total (of each type, if appropriate)?

The merged dataset consists of approximately 85,000 frames. For more information see Supplementary Figure S1.

Does the dataset contain all possible instances or is it a sample (not necessarily random) of instances from a larger set? If the dataset is a sample, then what is the larger set? Is the sample representative of the larger set (e.g., geographic coverage)? If so, please describe how this representativeness was validated/verified. If it is not representative of the larger set, please describe why not (e.g., to cover a more diverse range of instances, because instances were withheld or unavailable).

The merged dataset contains all possible instances from each individual source.

What data does each instance consist of? "Raw" data (e.g., unprocessed text or images) or features? In either case, please provide a description.

Each instance in the dataset comprises a side-view image featuring one or more animals. Accompanying these images are human annotated keypoints for each individual, which detail specific points of interest or markers on the animal's body. These keypoints provide valuable information for pose estimation and behavioral analysis.

Is there a label or target associated with each instance? If so, please provide a description.

The labels are the 2D coordinates (x, y in pixel space) and visibility flag (undefined, labeled, and unlabeled if occluded) per each keypoint for each dataset.

Is any information missing from individual instances? If so, please provide a description, explaining why this information is missing (e.g., because it was unavailable). This does not include intentionally removed information, but might include, e.g., redacted text.

Unknown to the authors of the merged dataset.

Are relationships between individual instances made explicit (e.g., users' movie ratings, social network links)? If so, please describe how these relationships are made explicit.

In the dataset of pictures or frames extracted from side-view videos of animals, the relationships between individual instances (frames) are not explicitly defined in terms of behavioral interactions or social links. Instead, the dataset primarily focuses on isolated frames as individual instances. Any temporal or behavioral relationships between the frames would be implicit, derived from the sequence in which they appear in the videos.

Are there recommended data splits (e.g., training, development/validation, testing)? If so, please provide a description of these splits, explaining the rationale behind them.

The dataset is partitioned into a train set, where individual datasets can be dropped to test OOD performance.

Are there any errors, sources of noise, or redundancies in the dataset? If so, please provide a description.

There are two primary sources of error in our dataset: firstly, annotation errors from the annotators of individual datasets may exist - we did not correct any original data source; and secondly, imperfections in the projection of keypoints from the original keypoint space to the target keypoint space cannot be guaranteed to not have occurred, although the authors did their best efforts to avoid such errors. Please see the preprocessing Methods section for more details and for the conversion table that the authors created.

Is the dataset self-contained, or does it link to or otherwise rely on external resources (e.g., websites, tweets, other datasets)? If it links to or relies on external resources, a) are there guarantees that they will exist, and remain constant, over time; b) are there official archival versions of the complete dataset (i.e., including the external resources as they existed at the time the dataset was created); c) are there any restrictions (e.g., licenses, fees) associated with any of the external resources that might apply to a future user? Please provide descriptions of all external resources and any restrictions associated with them, as well as links or other access points, as appropriate.

The merged dataset is self-contained and does not rely on external link that might change over time. Individual dataset links could be modified.

Does the dataset contain data that might be considered confidential (e.g., data that is protected by legal privilege or by doctor-patient confidentiality, data that includes the content of individuals non-public communications)? If so, please provide a description.

To our best knowledge, no such data is included.

Does the dataset contain data that, if viewed directly, might be offensive, insulting, threatening, or might otherwise cause anxiety? If so, please describe why.

Some images from iNaturalist contain dead rodents that could cause anxiety.

Does the dataset relate to people? If not, you may skip the remaining questions in this section.

No

Any other comments?

None.

Collection Process

How was the data associated with each instance acquired? Was the data directly observable (e.g., raw text, movie ratings), reported by subjects (e.g., survey responses), or indirectly inferred/derived from other data (e.g., part-of-speech tags, model-based guesses for age or language)? If data was reported by subjects or indirectly inferred/derived from other data, was the data validated/verified? If so, please describe how.

Individual datasets before merging were acquired from published papers or annotated by authors of the paper.
Datasets are validated and verified by the original dataset creators and later verified by the authors of this paper.

What mechanisms or procedures were used to collect the data (e.g., hardware apparatus or sensor, manual human curation, software program, software API)? How were these mechanisms or procedures validated?

No new data was collected for this merged dataset.

If the dataset is a sample from a larger set, what was the sampling strategy (e.g., deterministic, probabilistic with specific sampling probabilities)?

For publicly available data, please see their methods.

Who was involved in the data collection process (e.g., students, crowdworkers, contractors) and how were they compensated (e.g., how much were crowdworkers paid)?

We do not have information on the publicly available datasets. iRodent was annotated by Prof. Mackenzie Mathis and Tian Qiu at Harvard University and/or EPFL.

Over what time frame was the data collected? Does this time frame match the creation time frame of the data associated with the instances (e.g., recent crawl of old news articles)? If not, please describe the time frame in which the data associated with the instances was created.

Data was collected from 2020-2023.

Were any ethical review processes conducted (e.g., by an institutional review board)? If so, please provide a description of these review processes, including the outcomes, as well as a link or other access point to any supporting documentation.
Unknown.

Does the dataset relate to people? If not, you may skip the remaining questions in this section.
No.

Preprocessing/cleaning/labeling

Was any preprocessing/cleaning/labeling of the data done (e.g., discretization or bucketing, tokenization, part-of-speech tagging, SIFT feature extraction, removal of instances, processing of missing values)? If so, please provide a description. If not, you may skip the remainder of the questions in this section.
Data came from multiple sources and in multiple formats. To homogenize different annotation formats (COCO-style, DeepLabCut format, etc.), we implemented a generalized data converter. We parsed public datasets and reformatted them into a COCO-style project. Besides data conversion, the generalized data converter also implements key steps for the panoptic animal pose estimation task formulation, but no individual keypoints were changed.

Was the "raw" data saved in addition to the preprocessed/cleaned/labeled data (e.g., to support unanticipated future uses)? If so, please provide a link or other access point to the "raw" data.
The raw data was used, and can be extracted from the corresponding original source.

Is the software used to preprocess/clean/label the instances available? If so, please provide a link or other access point. Yes, the conversion table is available at https://github.com/AdaptiveMotorControlLab/modelzoo-figures.

Any other comments?
None.

Uses

Has the dataset been used for any tasks already? If so, please provide a description.
At the time of publication, only the original paper has used the dataset.

Is there a repository that links to any or all papers or systems that use the dataset? If so, please provide a link or other access point.
We suggest to check the citations of original paper sources.

What (other) tasks could the dataset be used for?
The dataset could be used for anything related to animal pose estimation.

Is there anything about the composition of the dataset or the way it was collected and preprocessed/cleaned/labeled that might impact future uses? For example, is there anything that a future user might need to know to avoid uses that could result in unfair treatment of individuals or groups (e.g., stereotyping, quality of service issues) or other undesirable harms (e.g., financial harms, legal risks) If so, please provide a description. Is there anything a future user could do to mitigate these undesirable harms?
Keypoint annotations from individual datasets were projected to a super-set keypoint space which represents this dataset. The model that is trained over this dataset might have bias on keypoints that are more common in the individual datasets and might have larger errors on keypoints that are under-represented in the source datasets.

Are there tasks for which the dataset should not be used? If so, please provide a description.
The dataset cannot be used to harm any animal. The dataset should not be used to train a model that is expected to be directly used (i.e., without further fine-tuning) for applications that require extremely high-precision, as there were annotator bias from the source datasets.

Any other comments?
None.

Distribution

Will the dataset be distributed to third parties outside of the entity (e.g., company, institution, organization) on behalf of which the dataset was created? If so, please provide a description.
Yes, the dataset will be publicly available with a license for use.

How will the dataset will be distributed (e.g., tarball on website, API, GitHub) Does the dataset have a digital object identifier (DOI)?
The dataset is distributed on zenodo (27).

When will the dataset be distributed?
The merged dataset is released with this paper).

Will the dataset be distributed under a copyright or other intellectual property (IP) license, and/or under applicable terms of use (ToU)? If so, please describe this license and/or ToU, and provide a link or other access point to, or otherwise reproduce, any relevant licensing terms or ToU, as well as any fees associated with these restrictions.
The data copyright belongs to the authors of the original datasets. Horse-30 is non-commercial user only.

Have any third parties imposed IP-based or other restrictions on the data associated with the instances? If so, please describe these restrictions, and provide a link or other access point to, or otherwise reproduce, any relevant licensing terms, as well as any fees associated with these restrictions.

Yes, please check the original sources. We assume no liability or guarantees on this model's use.

Do any export controls or other regulatory restrictions apply to the dataset or to individual instances? If so, please describe these restrictions, and provide a link or other access point to, or otherwise reproduce, any supporting documentation.
Unknown.

Any other comments?
None.

Maintenance

Who will be supporting/hosting/maintaining the dataset?
The dataset will be hosted on zenodo.

How can the owner/curator/manager of the dataset be contacted (e.g., email address)?
The head of The Mathis Lab of Adaptive Intelligence, Mackenzie Mathis, can be contacted at mackenzie.mathis@epfl.ch.

Is there an erratum? If so, please provide a link or other access point.
None.

Will the dataset be updated (e.g., to correct labeling errors, add new instances, delete instances)? If so, please describe how often, by whom, and how updates will be communicated to users (e.g., mailing list, GitHub)?
Not at this time.

If the dataset relates to people, are there applicable limits on the retention of the data associated with the instances (e.g., were individuals in question told that their data would be retained for a fixed period of time and then deleted)? If so, please describe these limits and explain how they will be enforced.
No.

Will older versions of the dataset continue to be supported/hosted/maintained? If so, please describe how. If not, please describe how its obsolescence will be communicated to users.
The data is banked on zenodo.

If others want to extend/augment/build on/contribute to the dataset, is there a mechanism for them to do so? If so, please provide a description. Will these contributions be validated/verified? If so, please describe how. If not, why not? Is there a process for communicating/distributing these contributions to other users? If so, please provide a description.
Others may do so and should contact the original authors about incorporating fixes/extensions.

Any other comments?
None.

Supplementary Discussion

Considerations on building general datasets for pretraining

To build generalizable pose models, a large-scale pre-training dataset is the key. It has been shown in both computer vision and natural language processing that pre-trained models significantly improve the generalization of models and data efficiency in the downstream tasks (28, 29). However, data of lab animals are not ubiquitous on the internet. To get large scale animal pose data, it is critical to gather the data directly from the research community in a responsible and transparent way. A platform that actively interacts with the community is thus required to build such a pre-training dataset. As such a vocabulary is built on top of a wide range of pose datasets, it can be used across different research needs and it is also key to for useful zero-shot inference (see Methods).

We acknowledge that these SuperAnimal models would not have been be possible without the accumulated data from the community. In the future, feedback from the community for models' efficacy and failure modes in different downstream data will be critical for updated model releases and algorithmic updates. As publicly available data increase, we expect the performance will improve.

Annotator bias in labeled data. Unlike previous works that require labeling data to create a working model, our models can be used as they are. For the purpose of evaluation, we could use the ground-truth of the target dataset or label frames of a novel video. We note that, when it comes to evaluating the performance of zero-shot inference, there will always be systematic errors between the model and the annotator of the target dataset. We refer to this type of error as annotator bias; i.e., annotators of different datasets try to place keypoints in slightly different places due to the bias of annotators. Therefore, the supervised metrics will tend to be an over-estimation of the error.

Conversely, SuperAnimal models can be used to monitor annotator bias as the model's predictions are consistent across frames while in many cases human annotators annotate keypoints in an inconsistent way.

Supervised metrics do not capture the richness of SuperAnimal-models

In pose estimation literature, work mostly report supervised metrics (RMSE, Normalized Error, and mAP). Common to them is that they do not penalize keypoints that are not annotated in the dataset. In contrast to other pose models, our SuperAnimal models can predict keypoints that are not annotated in the labeled dataset. For instance, if we apply only supervised metrics to evaluate SuperAnimal models, catastrophic forgetting is not detected as metrics do not penalize keypoint predictions that are not annotated.

Why we used a top-down approach for quadruped pre-trained pose models

Compared to the COCO keypoint benchmark (24), animal in the wild shows long tail distribution of subject sizes in both relative and absolute terms (Supplementary Figure S5a,b). As convolutional neural networks are not built to be scale-invariant, this can make it challenging for the models. Even though spatial-pyramid adaptation we proposed can mitigate it (Figure S5 c,d,e), early attempts show that bottom-up models give inferior performance compared to top-down models especially for quadruped data. Therefore, we chose top-down for quadruped as it standardized the animal the pose estimator sees, making the pre-training and test-time tasks easier.

How to use the DeepLabCut Model Zoo

The DeepLabCut Model Zoo consists of two parts. The first is a web-based platform that accepts pose data contributions, ranging from a DeepLabCut project, labeled images from our WebApp, and public animal pose datasets (See Figure S2d). As these data come in different formats, we implement a software-based data layer dubbed "generalized data converter" (see Methods) that convert data of various forms to DeepLabCut pose format. We call models we provide Super-Animal models for their generalization powers. After users download these super models from our website or via DeepLabCut APIs, they can either use the models as a plug-and-play solution or alternatively choose to adapt or fine-tune these models from videos or pose datasets.

Supplementary References

1. Jennifer J Sun, Markus Marks, Andrew Wesley Ulmer, Dipam Chakraborty, Brian Geuther, Edward Hayes, Heng Jia, Vivek Kumar, Sebastian Oleszko, Zachary Partridge, et al. Mabe22: a multi-species multi-task benchmark for learned representations of behavior. In *International Conference on Machine Learning*, pages 32936–32990. PMLR, 2023.
2. Jessy Lauer, Mu Zhou, Shaokai Ye, William Menegas, Steffen Schneider, Tanmay Nath, Mohammed Mostafizur Rahman, Valentina Di Santo, Daniel Soberanes, Guoping Feng, Venkatesh N. Murthy, George Lauder, Catherine Dulac, Mackenzie W. Mathis, and Alexander Mathis. Multi-animal pose estimation, identification and tracking with deeplabcut. *Nature Methods*, 19:496 – 504, 2022.

3. Oliver Sturman, Lukas von Ziegler, Christa Schläppi, Furkan Akyol, Mattia Privitera, Daria Slominski, Christina Grimm, Laetitia Thieren, Valerio Zerbi, Benjamin Grewe, et al. Deep learning-based behavioral analysis reaches human accuracy and is capable of outperforming commercial solutions. *Neuropsychopharmacology*, 45(11):1942–1952, 2020.
4. Lukas von Ziegler, Oliver Sturman, and Johannes Bohacek. Videos for deeplabcut, noldus ethovision X14 and TSE multi conditioning systems comparisons. https://doi.org/10.5281/zenodo.3608658. *Zenodo*, January 2020. doi: 10.5281/zenodo.3608658.
5. Isaac Chang. Trained DeepLabCut model for tracking mouse in open field arena with topdown view. https://doi.org/10.5281/zenodo.3955216. *Zenodo*, July 2020. doi: 10.5281/zenodo.3955216.
6. Simon RO Nilsson, Nastacia L. Goodwin, Jia Jie Choong, Sophia Hwang, Hayden R Wright, Zane C Norville, Xiaoyu Tong, Dayu Lin, Brandon S. Bentzley, Neir Eshel, Ryan J McLaughlin, and Sam A. Golden. Simple behavioral analysis (simba) – an open source toolkit for computer classification of complex social behaviors in experimental animals. *bioRxiv*, 2020. doi: 10.1101/2020.04.19.049452.
7. Alexander Mathis, Pranav Mamidanna, Kevin M Cury, Taiga Abe, Venkatesh N Murthy, Mackenzie Weygandt Mathis, and Matthias Bethge. Deeplabcut: markerless pose estimation of user-defined body parts with deep learning. *Nature neuroscience*, 21:1281–1289, 2018.
8. Jared M. Cregg, Roberto Leiras, Alexia Montalant, Paulina Wanken, Ian R. Wickersham, and Ole Kiehn. Brainstem neurons that command mammalian locomotor asymmetries. *Nature neuroscience*, 23:730 – 740, 2020.
9. Tanmay Nath, Alexander Mathis, An Chi Chen, Amir Patel, Matthias Bethge, and Mackenzie W Mathis. Using deeplabcut for 3d markerless pose estimation across species and behaviors. *Nature protocols*, 14:2152–2176, 2019.
10. Jingdong Wang, Ke Sun, Tianheng Cheng, Borui Jiang, Chaorui Deng, Yang Zhao, Dong Liu, Yadong Mu, Mingkui Tan, Xinggang Wang, et al. Deep high-resolution representation learning for visual recognition. *IEEE transactions on pattern analysis and machine intelligence*, 2020.
11. Shaoqing Ren, Kaiming He, Ross Girshick, and Jian Sun. Faster r-cnn: Towards real-time object detection with region proposal networks. *Advances in neural information processing systems*, 28, 2015.
12. Mu Zhou, Lucas Stoffl, Mackenzie W. Mathis, and Alexander Mathis. Rethinking pose estimation in crowds: overcoming the detection information-bottleneck and ambiguity. *IEEE/CVF International Conference on Computer Vision*, 2023.
13. Prianka Banik, Lin Li, and Xishuang Dong. A novel dataset for keypoint detection of quadruped animals from images. *ArXiv*, abs/2108.13958, 2021.
14. Jinkun Cao, Hongyang Tang, Haoshu Fang, Xiaoyong Shen, Cewu Lu, and Yu-Wing Tai. Cross-domain adaptation for animal pose estimation. *2019 IEEE/CVF International Conference on Computer Vision (ICCV)*, pages 9497–9506, 2019.
15. Daniel Joska, Liam Clark, Naoya Muramatsu, Ricardo Jericevich, Fred Nicolls, Alexander Mathis, Mackenzie W. Mathis, and Amir Patel. Acinoset: A 3d pose estimation dataset and baseline models for cheetahs in the wild. *2021 IEEE International Conference on Robotics and Automation (ICRA)*, pages 13901–13908, 2021.
16. Alexander Mathis, Thomas Biasi, Steffen Schneider, Mert Yuksekgonul, Byron Rogers, Matthias Bethge, and Mackenzie W Mathis. Pretraining boosts out-of-domain robustness for pose estimation. In *Proceedings of the IEEE/CVF Winter Conference on Applications of Computer Vision*, pages 1859–1868, 2021.
17. Aditya Khosla, Nityananda Jayadevaprakash, Bangpeng Yao, and Li Fei-Fei. Novel dataset for fine-grained image categorization. In *First Workshop on Fine-Grained Visual Categorization, IEEE Conference on Computer Vision and Pattern Recognition*, Colorado Springs, CO, June 2011.
18. Benjamin Biggs, Thomas Roddick, Andrew Fitzgibbon, and Roberto Cipolla. Creatures great and smal: Recovering the shape and motion of animals from video. In *Asian Conference on Computer Vision*, pages 3–19. Springer, 2018.
19. Hang Yu, Yufei Xu, Jing Zhang, Wei Zhao, Ziyu Guan, and Dacheng Tao. Ap-10k: A benchmark for animal pose estimation in the wild. In *Thirty-fifth Conference on Neural Information Processing Systems Datasets and Benchmarks Track (Round 2)*, 2021.
20. Yuxiang Yang, Junjie Yang, Yufei Xu, Jing Zhang, Long Lan, and Dacheng Tao. Apt-36k: A large-scale benchmark for animal pose estimation and tracking. *Advances in Neural Information Processing Systems*, 35:17301–17313, 2022.
21. iNaturalist. OGBIF Occurrence Download. https://doi.org/10.15468/dl.p7nbxt. *iNaturalist*, July 2020. doi: https://doi.org/10.15468/dl.p7nbxt.
22. Kaiming He, Georgia Gkioxari, Piotr Dollár, and Ross Girshick. Mask r-cnn. *Proceedings of the IEEE international conference on computer vision*, pages 2961–2969, 2017.
23. Tsung-Yi Lin, Piotr Dollar, Ross Girshick, Kaiming He, Bharath Hariharan, and Serge Belongie. Feature pyramid networks for object detection. In *Conference on Computer Vision and Pattern Recognition*, pages 936–944, 07 2017. doi: 10.1109/CVPR.2017.106.
24. Tsung-Yi Lin, Michael Maire, Serge J. Belongie, Lubomir D. Bourdev, Ross B. Girshick, James Hays, Pietro Perona, Deva Ramanan, Piotr Doll'a r, and C. Lawrence Zitnick. Microsoft COCO: common objects in context. *CoRR*, abs/1405.0312, 2014.
25. Microsoft. Cameratraps. `https://github.com/microsoft/CameraTraps`, 2023.
26. Mathis Laboratory of Adaptive Intelligence. Maushaus mathis lab. *Zenodo*, February 2024. doi: 10.5281/zenodo.10593101.
27. Mathis Laboratory of Adaptive Intelligence. Superanimal-quadruped-80k. *Zenodo*, February 2024. doi: 10.5281/zenodo.10619173.
28. Kaiming He, Xinlei Chen, Saining Xie, Yanghao Li, Piotr Dollár, and Ross Girshick. Masked autoencoders are scalable vision learners. In *Proceedings of the IEEE/CVF Conference on Computer Vision and Pattern Recognition*, pages 16000–16009, 2022.
29. Jacob Devlin, Ming-Wei Chang, Kenton Lee, and Kristina Toutanova. BERT: Pre-training of deep bidirectional transformers for language understanding. In Jill Burstein, Christy Doran, and Thamar Solorio, editors, *Proceedings of the 2019 Conference of the North American Chapter of the Association for Computational Linguistics: Human Language Technologies, Volume 1 (Long and Short Papers)*, pages 4171–4186, Minneapolis, Minnesota, June 2019. Association for Computational Linguistics. doi: 10.18653/v1/N19-1423.
