# Supplementary material for: SuperAnimal pretrained pose estimation models for behavioral analysis
Source: Nat Commun. 2024 Jun 21;15:5165. doi: 10.1038/s41467-024-48792-2 (PMC11192880; doi:10.1038/s41467-024-48792-2)
Supplement: Supplementary file 3 — Description of Additional Supplementary Files [file 41467_2024_48792_MOESM3_ESM.pdf]

Description of Additional Supplementary Files

File name: Supplementary Movie 1
Description: Video prediction results by comparison model trained with and without gradient masking.

File name: Supplementary Movie 2
Description: Video prediction results by SuperAnimal model fine-tuned with naive-fine-tuning and SuperAnimal model fine-tuned with memory replay.

File name: Supplementary Movie 3
Description: Video prediction results by SuperAnimal-TopViewMouse model with and without spatial pyramid inference. Note that because we use a detector for the SuperAnimal-Quadruped, this is not needed.

File name: Supplementary Movie 4
Description: Video prediction results by SuperAnimal models with and without video adaptation.

File name: Supplementary Movie 5
Description: Example video from Sturman et al (15) vs. SuperAnimal-TopViewMouse without any training.

File name: Supplementary Movie 6
Description: Top-down based SuperAnimal-TopviewMouse's video prediction from one example MABe video, without being trained on any MABe videos.
