# Supplementary material for: SuperAnimal pretrained pose estimation models for behavioral analysis
Source: Nat Commun. 2024 Jun 21;15:5165. doi: 10.1038/s41467-024-48792-2 (PMC11192880; doi:10.1038/s41467-024-48792-2)
Supplement: Supplementary file 10 — Reporting Summary [file 41467_2024_48792_MOESM10_ESM.pdf]

nature portfolio

Corresponding author(s): Mackenzie Mathis

Last updated by author(s): 2024-Jan-30

Reporting Summary

Nature Portfolio wishes to improve the reproducibility of the work that we publish. This form provides structure for consistency and transparency in reporting. For further information on Nature Portfolio policies, see our Editorial Policies and the Editorial Policy Checklist.

Statistics

For all statistical analyses, confirm that the following items are present in the figure legend, table legend, main text, or Methods section.

n/a	Confirmed	
☐	☒	The exact sample size (*n*) for each experimental group/condition, given as a discrete number and unit of measurement
☐	☒	A statement on whether measurements were taken from distinct samples or whether the same sample was measured repeatedly
☐	☒	The statistical test(s) used AND whether they are one- or two-sided<br>*Only common tests should be described solely by name; describe more complex techniques in the Methods section.*
☐	☒	A description of all covariates tested
☐	☒	A description of any assumptions or corrections, such as tests of normality and adjustment for multiple comparisons
☐	☒	A full description of the statistical parameters including central tendency (e.g. means) or other basic estimates (e.g. regression coefficient) AND variation (e.g. standard deviation) or associated estimates of uncertainty (e.g. confidence intervals)
☐	☒	For null hypothesis testing, the test statistic (e.g. *F*, *t*, *r*) with confidence intervals, effect sizes, degrees of freedom and *P* value noted<br>*Give P values as exact values whenever suitable.*
☒	☐	For Bayesian analysis, information on the choice of priors and Markov chain Monte Carlo settings
☒	☐	For hierarchical and complex designs, identification of the appropriate level for tests and full reporting of outcomes
☐	☒	Estimates of effect sizes (e.g. Cohen's *d*, Pearson's *r*), indicating how they were calculated

Our web collection on statistics for biologists contains articles on many of the points above.

Software and code

Policy information about availability of computer code

Data collection	MausHaus: We collected video data from five single-housed C57BL/6J male and female mice in an extended home cage, carried out in the laboratory of Mackenzie Mathis at Harvard University and also EPFL (temperature of housing was 20-25C, humidity 20-50%). Data were recorded at 30Hz with 640 x 480 pixels resolution acquired with White Matter, LLC eV cameras. Annotators localized 26 keypoints across 322 frames sampled from within DeepLabCut using the k-means clustering approach. All experimental procedures for mice were in accordance with the National Institutes of Health Guide for the Care and Use of Laboratory Animals and approved by the Harvard Institutional Animal Care and Use Committee (IACUC) (n=l mouse), and by the Veterinary Office of the Canton of Geneva (Switzerland; license GEOl) (n= 4 mice). Further details will be published elsewhere<br>Data analysis
Data analysis	Python3 with DeepLabCut or mmpose was used. All new custom code is available at deeplabcut.org as of version 2.3.1 (March 2023) to Jan 2024 (2.3.8).

For manuscripts utilizing custom algorithms or software that are central to the research but not yet described in published literature, software must be made available to editors and reviewers. We strongly encourage code deposition in a community repository (e.g. GitHub). See the Nature Portfolio guidelines for submitting code & software for further information.

Data

Policy information about availability of data

All manuscripts must include a data availability statement. This statement should provide the following information, where applicable:

- Accession codes, unique identifiers, or web links for publicly available datasets
- A description of any restrictions on data availability
- For clinical datasets or third party data, please ensure that the statement adheres to our policy

We collected publicly available datasets from the community, as well as in-house datasets for building the SuperAnimal models. Thereby, we sought to cover diverse individuals, backgrounds, scenarios, and postures. In the following we detail the references for those datasets:

TopViewMouse-5k. 3CSI, BM, EPM, LDB, OFT See full details at (15) and in (57). BlackMice See full details at (24). WhiteMice See details in SIMBA (25). Courtesy of Prof. Sam Golden and Nastacia Goodwin. TriMouse See full details at (10). DLC-Openfield See full details at (9). Kiehn-Lab-Openfield, Swimming, and treadmill See details at (58). Courtesy of Prof. Ole Kiehn, Dr. Jared Cregg, and Prof. Carmelo Bellardita. MausHaus We collected video data from five single-housed C57BL/6J male and female mice in an extended home cage, carried out in the laboratory of Mackenzie Mathis at Harvard University and also EPFL (temperature of housing was 20-25C, humidity 20-50%). Data were recorded at 30Hz with 640 × 480 pixels resolution acquired with White Matter, LLC eV cameras. Annotators localized 26 keypoints across 322 frames sampled from within DeepLabCut using the k-means clustering approach (59). All experimental procedures for mice were in accordance with the National Institutes of Health Guide for the Care and Use of Laboratory Animals and approved by the Harvard Institutional Animal Care and Use Committee (IACUC) (n=1 mouse), and by the Veterinary Office of the Canton of Geneva (Switzerland; license GE01) (n=4 mice). MausHaus data is banked on zenodo (60).
For ease of use, we packaged these datasets into one directory that can be accessed at https://zenodo.org/records/10618947 (61).

Quadruped-80K. AwA-Pose Quadruped dataset, see full details at (62). AnimalPose See full details at (28). AcinoSet See full details at (26). Horse-30 Horse-30 dataset, benchmark task is called Horse-10; See full details at (16). StanfordDogs See full details at (63, 64). AP-10K See full details at (31). APT-36K See full details at (32) iRodent We utilized the iNaturalist API functions for scraping observations with the taxon ID of Suborder Myomorpha (65). The functions allowed us to filter the large amount of observations down to the ones with photos under the CC BY-NC creative license. The most common types of rodents from the collected observations are Muskrat (Ondatra zibethicus), Brown Rat (Rattus norvegicus), House Mouse (Mus musculus), Black Rat (Rattus rattus), Hispid Cotton Rat (Sigmodon hispidus), Meadow Vole (Microtus pennsylvanicus), Bank Vole (Clethrionomys glareolus), Deer Mouse (Peromyscus maniculatus), White-footed Mouse (Peromyscus leucopus), Striped Field Mouse (Apodemus agrarius). We then generated segmentation masks over target animals in the data by processing the media through an algorithm we designed that uses a Mask Region Based Convolutional Neural Networks(Mask R-CNN) (66) model with a ResNet-50-FPN backbone (45), pretrained on the COCO datasets (40). The processed 443 images were then manually labeled with pose annotations, and bounding boxes were generated by running Mega Detector (67) on the images, which were then manually verified. iRodent data is banked at https://zenodo.org/record/8250392.
For ease of use, we packaged these datasets into one directory, which is banked at: https://zenodo.org/records/10619173 (68).

Additional OOD Videos. In Figure 3, for video testing we additionally used the following data: Golden Lab mouse: see details at (69). Smear Lab Mouse: see details at (70). Mathis Lab MausHaus: New video conditions, but the same as MausHaus ethics approval as above. BlackDog: video from https://www.pexels.com/video/unleashing-the-pet-dog-outdoors-4763071/, Elk video from https://www.pexels.com/video/a-deer-looking-for-food-in-the-ground-covered-with-snow-3195531/. Horse-30 videos: we used the ground truth annotations for 30 horse videos as described (16).

The Two SuperAnimal datasets generated in this study have been deposited in the Zenodo database under DOIs (61, 68). These packaged datasets are detailed in the “Datasheets” in Supplementary Information. The SuperAnimal model weights are banked at HuggingFace: https://huggingface.co/mwmathis/DeepLabCutModelZoo-SuperAnimal-Quadruped and https://huggingface.co/mwmathis/DeepLabCutModelZoo-SuperAnimal-TopViewMouse, and see the detailed “Model Cards” in Supplementary Information.

Code to use the DeepLabCut Model Zoo: https://github.com/DeepLabCut/DeepLabCut; it is available since version 2.3.1 and at the time of final acceptance the code is at version 2.3.9. Code and data to reproduce the figures: https://github.com/AdaptiveMotorControlLab/modelzoo-figures. All other requests should be made to the corresponding author.

Research involving human participants, their data, or biological material

Policy information about studies with human participants or human data. See also policy information about sex, gender (identity/presentation), and sexual orientation and race, ethnicity and racism.

Reporting on sex and gender	N/A
Reporting on race, ethnicity, or other socially relevant groupings	N/A

Population characteristics	N/A
Recruitment	N/A
Ethics oversight	N/A

Note that full information on the approval of the study protocol must also be provided in the manuscript.

Field-specific reporting

Please select the one below that is the best fit for your research. If you are not sure, read the appropriate sections before making your selection.

☒ Life sciences ☐ Behavioural & social sciences ☐ Ecological, evolutionary & environmental sciences

For a reference copy of the document with all sections, see nature.com/documents/nr-reporting-summary-flat.pdf

Life sciences study design

All studies must disclose on these points even when the disclosure is negative.

Sample size	No sample size was predetermined, but exceeds standard animal pose estimation datasets. Namely, the typical prior datasets were 100-8000 frames, and here we provide of 85K. Dataset size was tested in the manuscript and number of frames required for a given performance level is reported.
Data exclusions	No data was excluded.
Replication	Models were trained with multiple seeds and all reported. Moreover, we validated our results in Extended Data Fig. 3 with different permutations of the input data, and in Figures 1 & 2 with different neural networks
Randomization	This is not relevant to our study as no data was explicitly excluded during training aside from a forming dedicated train and test sets as is standard in the field of machine learning.
Blinding	Blinding is not relevant as there is no control group.

Reporting for specific materials, systems and methods

We require information from authors about some types of materials, experimental systems and methods used in many studies. Here, indicate whether each material, system or method listed is relevant to your study. If you are not sure if a list item applies to your research, read the appropriate section before selecting a response.

Materials & experimental systems

n/a	Involved in the study	
☒	☐	Antibodies
☒	☐	Eukaryotic cell lines
☒	☐	Palaeontology and archaeology
☐	☒	Animals and other organisms
☒	☐	Clinical data
☒	☐	Dual use research of concern
☒	☐	Plants

Methods

n/a	Involved in the study	
☒	☐	ChIP-seq
☒	☐	Flow cytometry
☒	☐	MRI-based neuroimaging

Animals and other research organisms

Policy information about studies involving animals; ARRIVE guidelines recommended for reporting animal research, and Sex and Gender in Research

Laboratory animals	5 C57BLK6/J mice of female/male sex were used. Mice were housed in an inverted light cycle room at 25C 12h/12h.
Wild animals	No primary raw data was collected from wild animals, only publicly available images
Reporting on sex	Sex was not a factor (only 5 mice used), and sex was roughly equal.
Field-collected samples	N/A
Ethics oversight	All experimental procedures for mice were in accordance with the National Institutes of Health Guide for the Care and Use of

Ethics oversight	Laboratory Animals and approved by the Harvard Institutional Animal Care and Use Committee (IACUC) (n=l mouse), and by the Veterinary Office of the Canton of Geneva (Switzerland; license GE0l) (n=4 mice)

Note that full information on the approval of the study protocol must also be provided in the manuscript.

Plants

Seed stocks	N/A
Novel plant genotypes	N/A
Authentication	N/A
